# Effectiveness of childhood pneumonia awareness program among pregnant women: A hospital based quasi experimental study

**DOI:** 10.1371/journal.pone.0342216

**Published:** 2026-11-06

**Authors:** Sushmita Ghimire, Padma Shrestha, Nani Maiya Kaway, Bijay Bajracharya, Deena Shrestha

**Affiliations:** 1 Department of Epidemiology and public health, Center for Health and Disease Studies Nepal, Kathmandu, Nepal; 2 Nursing Department, Paropakar Maternity and Women’s Hospital, Kathmandu, Nepal; Group for Technical Assistance / Asian College for Advance Studies, Purbanchal University, NEPAL

## Abstract

**Background:**

Pneumonia poses a substantial threat to the lives of the under five children in Nepal, underscoring the crucial role of maternal awareness in preventing this condition. The objective of the program was to assess the effectiveness of the Pneumonia awareness program designed to educate the expectant mothers, enrich their understanding, and empower them to take timely preventive measures against the Pneumonia.

**Methods:**

The study utilized an intervention-based concurrent mixed methods design, employing a quasi-experimental study to evaluate awareness and an exploratory design to assess perception of the pregnant women attending the hospital. A structured questionnaire was used for quantitative data, and interview guideline for qualitative study to conduct face-to-face interviews. The data analysis was performed by using STATA software. The knowledge of pneumonia before and after the awareness campaign was compared using a paired t test. The structured questionnaire covered a number of areas pertaining to pneumonia, including an overview of the disease, its symptoms, its mode of transmission, vaccination, and risk factors. The STROBE guideline was used to ensure standard reporting of the results.

**Results:**

The findings revealed the enhancement in the participant’s knowledge. Their skill in identifying symptoms and immunizing their children against pneumonia improved. Notably, there was significant change in the knowledge level from pretest score to post test score indicating the effectiveness of the educational intervention. The participants in qualitative findings showed improved health –seeking behaviour, increased awareness of pneumonia and their commitment to preventive measures. The proactive intentions of early health seeking practice through identification of danger signs and adopting the preventive measures as complete dose of vaccination and controlling other risk factors were conveyed by the participants. Also, they perceived the program as effective in enhancing their knowledge and promoting their healthy behaviour.

**Conclusion:**

The findings highlight the program's effectiveness and emphasize the importance of regularly promoting similar awareness campaigns as a strategy to improve maternal and child health. Educational programs play a crucial role in empowering mothers to take timely preventive measures, thereby reducing pneumonia-related risks in children under five. Regular promotion of such campaigns is recommended to maintain and enhance maternal and child health.

## Background

Childhood Pneumonia stands as a significant cause of illness and death among children under five globally, with a pronounced impact in low and middle income countries [1]. Neonatal mortality in these regions contributes to 50–60% of infant deaths, with acute respiratory infections posing the highest mortality risk in the first two months of life [2]. Approximately one-fifth of all deaths in children under five result from pneumonia, with over 70% occurring in sub-Saharan Africa and southeast Asia [3,4]. Despite notable progress in child health in Nepal, pneumonia ranks among the top five diseases causing child mortality, affecting 2.1% of children under five [5]. According to annual report 2024, the national pneumonia incidence rate among children under five years was 55.1 per 1000 with 22 deaths attributed to acute respiratory infections [6]. A study at the tertiary hospitals of Nepal at Kathmandu valley showed 60.8% of children had acute respiratory infection (ARI) among which 14.3 had severe or very severe pneumonia [7]. However, with early detection, pneumonia can be treated easily and affordably using readily available medication [8].

Nepal has set a target under the Sustainable Development Goals to lower under-five mortality to 25 deaths per 1,000 live births by 2030 [9]. In order to address childhood pneumonia, Nepal has commenced a number of public health initiatives, primarily led by the Ministry of Health and Population (MoHP) and involving various national and international collaborators or partners [10]. These initiatives heavily rely on the National Health Education, Information and Communication Center (NHEICC), an apex body for development and dissemination of health education materials and resources aiming at improving community health literacy and practices [11]. Moreover, the introduction and promotion of the pneumococcal conjugate vaccine in the national immunization program, aiming to reduce the morbidity and mortality from childhood pneumonia, further demonstrates the government's commitment and dedication to the cause [12].

Despite these continued efforts, pregnant women still have substantial knowledge gap due to which 12.3% of the children with pneumonia in Nepal did not seek any treatment for pneumonia [13]. Evidence indicates that one in ten children with pneumonia did not receive any treatment [6,13]. Several studies in Nepal and India revealed that only less than half of the mothers recognized the signs and symptoms of ARI, emphasizing the need to educate mothers to improve their understanding on cause, symptoms and treatment through different intervention like awareness program [14–16]. Early detection and referral play a crucial role in minimizing pneumonia fatalities in children, and this process relies on recognizing symptoms at home and promptly seeking medical attention [17,18]. Mothers, being primary caretakers, play a pivotal role in a child's health, necessitating comprehensive awareness of pneumonia signs, symptoms, and prevention measures [19,20].

Studies show a clear link between mothers’ knowledge levels regarding pneumonia symptoms and infection rates among children, as well as a connection between poor symptom recognition and delayed health-seeking behavior [21–24].

Given the pivotal role of mothers in child care, it is imperative to provide them with essential information about pneumonia during antenatal visits. Existing literature highlights that targeted educational interventions can effectively enhance knowledge and positively impact health behaviors. For example: an evidence from Bangladesh demonstrate the increased knowledge about pneumonia and other childhood illness due to community based maternal education program leading to better health service seeking behaviours and reduced child mortality [25]. Likewise, studies conducted in both community and hospital settings in India demonstrated significant improvements in maternal knowledge following the implementation of educational programs [16,26]. These educational program targeting mothers not only equipped them with basic knowledge about pneumonia but also serve as a means to convey disease-related messages to the wider community [25,26]. In addition, an intervention conducted through home visits in Indonesia demonstrated an increase in maternal knowledge, promoting self-efficacy, self-management, and heightened awareness for ARI prevention [27]. In Nepal, there is limited yet encouraging evidence on the effectiveness of community-based educational interventions in enhancing maternal knowledge. A pilot intervention that incorporated health education for mothers in rural communities to address ARI demonstrated a significant reduction in ARI-related mortality [28]. Similarly, a community based initiative conducted across four districts in Nepal, which employed various methods to disseminate health messages about ARI, showed a moderate impact, highlighting its potential to drive large-scale behavioral changes [29]. However, there is a limited focus on interventions addressing childhood pneumonia awareness among mother in Nepal. The opportunity to deliver health education during perinatal contact with mothers, particularly in urban areas, further emphasizes the need for interventions [2].

This study seeks to address two key research questions: whether a structured educational intervention on childhood pneumonia can enhance the knowledge of expectant mothers at Paropakar Maternity and Women’s Hospital in Kathmandu, Nepal, and which specific aspects of maternal knowledge, such as understanding pneumonia symptoms, preventive measures, and care-seeking behaviors, are most influenced by the intervention.

The objectives of this study are to assess the effectiveness of the educational intervention and to measure the change in maternal knowledge due to the educational intervention about childhood pneumonia among expectant mothers attending Paropakar Maternity and Women’s Hospital in Kathmandu, Nepal. This intervention is intended to supplement current government initiatives by utilizing NHEICC resources and emphasizing important topics, including the significance of immunization, identifying pneumonia symptoms, and explaining preventive measures. By assessing the effectiveness of the Childhood Pneumonia Awareness Program, this study aims to contribute to improved child health outcomes in Nepal and inform future maternal education initiatives in similar contexts.

## Methods

### Study design

An intervention based concurrent mixed method was applied in this study [30], as it allows for the simultaneous assessment of the intervention’s measurable outcomes and an in-depth exploration of mothers’ perspectives on its effectiveness. This dual approach ensures that the findings are not only statistically significant but also meaningful and actionable within the local context. A quasi-experimental study design [31] was carried out to evaluate the awareness among pregnant women attending the hospital and exploratory descriptive study design was used for qualitative study [32]. Since, it was difficult to implement the randomization in the real –world setting without interfering with their routine health care, logistical and ethical issues prevented randomization from being used. Additionally, the study also sought to ensure that the intervention was provided to all the eligible pregnant women, upholding equity and avoiding potential ethical dilemmas that might arise from the some participants not benefiting from important health information. A single group pre-post study design was applied for quantitative design and semi structured interview approach for qualitative design. In order to provide a comprehensive evaluation of the educational program, a descriptive exploratory study design with inductive thematic analysis was implemented in order to acquire a comprehensive understanding of participants’ experiences, capture contextual factors impacting the effectiveness of the intervention, obtain detailed feedback, and strengthen the validity of findings through triangulation.

### Conceptual and operational definitions

#### Awareness.

Conceptually, awareness refers to the state or capacity of perceiving, recognizing, or being conscious of the existence, characteristics, and significance of something, including events, objects, thoughts, or sensory experiences [33–35]. In this study, awareness refers to the broader recognition and understanding of childhood pneumonia, including its signs and symptoms, risk factors, preventive measures, and appropriate care-seeking practices. Awareness was considered a broader construct and was explored qualitatively across these key aspects of childhood pneumonia.

#### Knowledge.

Conceptually, knowledge refers to factual information, understanding, and skills acquired through education, discovery, learning, or experience [35–37]. In this study, knowledge represents the factual and measurable component of awareness related to childhood pneumonia. Operationally, knowledge was defined as participants’ ability to correctly identify and respond to questions concerning childhood pneumonia, including its signs and symptoms, modes of transmission, vaccination, and risk factors. Knowledge was considered a specific and measurable component of the broader construct of awareness and was assessed quantitatively using a structured pre-test and post-test questionnaire.

#### Pneumonia.

Pneumonia is an acute respiratory infection primarily caused by viral or bacterial pathogens. Although it can affect individuals of all ages and range from mild to severe, potentially life-threatening illness, it remains the leading infectious cause of death among children globally [38]. In this study, pneumonia refers to the pneumonia among the children as described in WHO guidance and presented about it’s general overview, signs and symptoms, mode of transmission, risk factors, prevention and appropriate care seeking in the educational intervention.

#### Awareness program.

Conceptually, an awareness program refers to an organized communication activity designed to increase awareness and understanding of a particular topic among a target population, with the aim of encouraging appropriate behavioural change and improving desired outcomes [39]. It can also be considered an approach to health education which comprises the learning experiences designed to improve health by increasing knowledge, influencing motivation and improving health literacy [40]. In this study, an awareness program refers to a planned impersonal health education and information sessions intended to increase participants’ recognition and understanding of childhood pneumonia using audio visual media.

#### Educational intervention.

Conceptually, an educational intervention refers to a planned and structured activity implemented by an external agent, such as a teacher, educator, or researcher, with the intention of influencing or improving learners’ knowledge, cognitions, emotions, attitudes, or behaviours [41]. Educational intervention in this study explains the structured educational sessions or the package of awareness program delivered to the pregnant women by the researcher. It covered an overview of pneumonia, childhood pneumonia, and mode of transmission, signs and symptoms of pneumonia, pneumonia vaccines, risk factors, and preventive measures.

#### Effectiveness of education intervention.

Conceptually, the effectiveness of an educational intervention refers to the extent to which a planned educational intervention achieves its intended outcomes among the target population. These outcomes may include improvements in knowledge, understanding, skills, attitudes, confidence, or health-related behaviours, depending on the objectives of the intervention [42,43]. Effectiveness of education intervention in this study was primarily operationalized as the change in participants’ knowledge scores between the pre-test and post-test assessments following the educational intervention. Participants’ self-reported perception of having gained knowledge or awareness following the intervention was considered a complementary measure of perceived benefit rather than the primary measure of effectiveness.

### Intervention

An educational intervention was designed to improve the knowledge on pneumonia among pregnant women attending ANC clinic. The pregnant women attending ANC Outpatient Department (OPD) clinics were invited for an educational session that lasted for 30 minutes. A pre-test questionnaire was administered before starting to the educational session. Considering that, there could be illiterate participants in the study, for those participants the questions in the pre-test questionnaire is asked by the one of the researcher to collect the information. The educational content was primarily developed based in the existing national and international guidelines. The contents included the information from the World Health Organization (WHO) and NHEICC. The content on the educational or informative session with audio-visual presentation covered an overview of pneumonia, childhood pneumonia, and mode of transmission, signs and symptoms of pneumonia, pneumonia vaccines, risk factors, and preventive measures. The audio-visual aids were prepared in the Nepalese language to ensure that all pregnant women, both literate and illiterate, received the knowledge. A panel of public health experts, microbiologist and health specialist as Senior Nurses reviewed the materials to ensure accuracy and relevance of the content to disseminate. The content was finalized after incorporating feedback from content experts and was subsequently used as the educational intervention that was delivered to expectant women attending the OPD. The educational intervention was delivered through face to face by the public health professionals experiencing in health education activities in Nepali language. A hall provided by the hospitals was used as a setting to deliver this educational intervention. Following the sessions, participants were given additional time for the question-and-answer sessions. After resolving their queries, a post test questionnaire was administered. The educational program was continued for the five days during Pneumonia week of action (Nov 7–12, 2022). Despite the educational sessions reaching thousands of pregnant women attending OPD at hospitals, our evaluation of the program was conducted only on a specified sample necessary for the assessment. Along with the audio-visuals and presentations, educational brochures printed by government were also distributed to the participants so that they can learn in the future. Example of educational material is provided in S1 File.

### Study site

Study site was the OPD of Antenatal clinic of Paropakar Maternity and Women’s hospital which is the only public tertiary level hospital in Kathmandu city with high case load, delivering only reproductive and sexual health related services. Thus, this site was ideal for delivering Pneumonia awareness program among pregnant women.

### Sample size

The sample size was calculated by using the following parameters.

Assumed proportion of the pregnant women knowing disease (P_1_)= 50% The expected improvement in knowledge level was assumed to be 30%. Proportion of knowledge expected after intervention (P_2_) =80%

Using a standard formula for comparing two proportions, with a two-sided α of 5% and 80% power; significance level (α) = 5% and Power (β) = 80% [44].


$$\begin{array}{c} n=\frac{{(z_{\frac{\alpha }{2}}+z_{\beta })}^{2\;}[P_{1}\left(1-P_{1}\right)+P_{2}\left(1-P_{2}\right)]}{{(P_{1}-P_{2})}^{2}}=\text{\textasciitilde{}}35.72\text{\textasciitilde{}per\textasciitilde{}group} \end{array}$$

The minimum required sample size was estimated as 35.72, which was rounded to 36 participants per group. Allowing for a 20% attrition rate, the required sample size was increased to approximately 45 participants per group (90 participants in total). As the intervention was carried out for 5 days long, so the total participant estimated to reach 450 participants (90*5).

Hence, the sample size was estimated considering the following parameters and sample size was estimated to be 450 participants. In total 508 participants participated in the study and eleven In-depth Interviews were conducted after awareness program guided by the data saturation theory [45].

### Sampling technique

The study incorporated pregnant women who were part of the pneumonia awareness program. Educational sessions were held within a training facility made available by Paropakar Maternity and Women’s Hospital. A total of 50 sessions took place over five days, with 25 sessions selected for the pre-post assessment. Simple random sampling was used to determine which sessions would undergo the pre-post-test, and all women enrolled in those selected sessions became part of the study. The average session participation ranged from 10 to 25 participants. Consequently, the total number of women included in the pre-post-test analysis account to 508.

The method employed for participant selection in qualitative in-depth interviews was purposive judgmental sampling [46]. Enumerators exercised their own judgment when selecting individuals for the survey. The criteria for participant selection included attendance at the educational session on childhood pneumonia, availability to participate in the survey or qualitative interviews, and willingness to share their viewpoints and experiences regarding the intervention. The selection process was conducted transparently, with deliberate efforts to minimize selection bias by ensuring diversity among participants to capture a wide range of perspectives. Qualitative interviews concluded upon reaching data saturation.

### Data collection tools and techniques

The participants for the study were recruited from November 7 2022 to November 12 2022 during the Pneumonia week of action. The structured questionnaire designed for this study encompasses socio-demographic data, pre-post test questions, and qualitative interview guideline focused on assessing knowledge, attitudes toward pneumonia, and evaluating the effectiveness of the awareness program. The questionnaire developed was reviewed by the content experts to ensure its content validity and cultural relevance. Additionally, the questionnaire was pre-tested before administration for local validity and reliability. The questionnaire was pretested with a sample of 50 pregnant women who were not part of the main study and shared the similar characteristics with the target population. The Cronbach’s alpha value obtained from the pre tested data was 0.78 which is considered as an acceptable internal reliability [47]. Only minor language adjustments were made based on feedback received from experts and participants during finalization process.

A process of providing the information and obtaining the consent was carried out before enrolling the study participants for the evaluation. The structured questionnaire for the quantitative study and interview guidelines for qualitative study were applied for data collection. (S2 File) The face-to-face interview was the data collection technique. The answers provided by the research participants are maintained privacy and kept confidentially.

### Data management and analysis

All completed survey forms were manually revised and coded before being entered into an excel spread-sheet. Using STATA 13 2022, data analysis was carried out after cleaning in Excel. A McNemar’s test was used to compare proportion change in correct knowledge about pneumonia before and after the awareness campaign. Each participant's response to each question was dichotomised as correct or incorrect knowledge. McNemar’s test was applied using individual-level paired responses for each question, although only aggregated proportions are presented in the table for presentation purposes. Changes in participants’ knowledge scores from before to after the awareness session were analysed using a paired t-test. The difference scores were checked for approximate normality before applying the test. The questionnaire for the knowledge on pre- and post-test included a total of 14 questions. The queries were organized into domains. i) Introduction to pneumonia (Q.1 and Q.2) ii) symptoms of pneumonia, (Q3) iii) mode of transmission, (Q4 and Q5) iv) information on vaccines, (Q6, Q7, Q8, Q9 and Q10) and v) risk factors (Q11, Q12, Q13 and Q14). A bivariate analysis was conducted to measure the relationship between the knowledge difference as dependent variable and socio demographic characteristics as an independent variable individually. Linear regression analysis was conducted with their corresponding 95% confidence intervals (CI). The p-values from this first bivariate analysis were then evaluated, and variables with p-values less than 0.25 were taken into consideration for inclusion in the multivariable model. The assumptions of multiple linear regression were assessed before fitting the final model. The assumptions of multiple linear regression were adequately met, with normally distributed residuals (Shapiro–Wilk W = 0.996, p = 0.163), homoscedasticity (Breusch–Pagan/Cook–Weisberg test: χ^2^(1) = 0.01, p = 0.925), Notably the VIF values for all variables in our analysis were less than two. As a result, the final multivariable linear regression model included all relevant variables and fixed the significance level at 5%.

For Qualitative data analysis, the data collected from the interview were translated. The translated documents were used for the analysis of the data. Inductive thematic analysis was conducted [48], during which the English-language transcripts were verified by the researchers, and codes were created from the documents. Two authors independently coded the translated interview texts to ensure intercoder reliability. The coding process was carried out by two coders, with all codes from the second coder aligning with those from the first. Any discrepancies in the codes were collaboratively reviewed and discussed among the authors, and the findings were presented based on their relevance. Common codes were grouped, and those with similar meanings were combined. Broader themes were then developed based on the generated codes within the framework of the basic themes. The trustworthiness of the data was ensured through conformability, credibility, dependability, and transferability [48]. Confidentiality and anonymity of the research participants were maintained at all stages of the research process.

Following this, the triangulation of the results obtained from both qualitative and quantitative data was done to increase the validity of the findings [49]. Findings from both components demonstrated convergence across all assessed areas, with qualitative responses consistently supporting the quantitative results. No divergent or contradictory findings were identified.

### Ethical consideration

The research protocol was approved by Institutional Review Committee at Paropakar Maternity and Women’s Hospital (Reference Number: 63/428). Written informed consent was obtained from all the literate participants and thumb prints of illiterate participants were taken after reading out the consent form before the interview. The study adhered to the ethical review guidelines of the Nepal Health Research Council. Participants were fully informed about the research, including its significance, potential benefits and harms, and their right to withdraw from the study at any time without giving any reason or justification. Ethical considerations were carefully considered to uphold fairness, respect for participants, and the integrity of the research process. Participants with prior knowledge of pneumonia could potentially influence the intervention's outcomes. To mitigate this, all participants received same educational materials and sessions to ensure consistency and inclusivity. Additionally, pre-intervention assessments were conducted to account for differences in baseline knowledge, allowing for accurate measurement of knowledge changes following the intervention.

## Results

### Background characteristics of the study participants

The average age of the participants was 26.42y ± 4.66y. The participants were from various castes and ethnicity; with Janajati being the largest group (48.03%), followed by Brahmins/Chhetri (38.9%) and Dalits (8.3%). The majority of the respondents were Hindus (72.8%), followed by Buddhists (19.3%), Christians (5.5%), Islam (1.4%) and Kirat (0.9%). On average, the family size of the participants was 4.59 ± 2.19, and more than half of the respondents lived in nuclear families (55.3%). More than half of the respondents (54.5%) were at their first pregnancy. Educational background of the participants varied, with majority completing secondary school (35.0%) and fewer being illiterate (5.7%). Employment status of the participants also varied, with majority being homemakers (48.2%). Majority of the participants lived in urban areas (85.0%), and more than half had experience in child caring (65.75%) (Table 1).

**Table 1:** The socio-demographic characteristics of the study participants (N = 508).

Variables	Characteristics	Frequency	Percentage
**Mean Age**	26.42 + 4.66		
**Caste/ Ethnicity**	Brahmin /Chhetri	198	38.9
	Madhesi	18	3.5
	Dalits	42	8.3
	Janajati	244	48.0
	Muslims	5	0.9
	Others	1	0.2
**Religion**	Hindu	370	72.8
	Buddhist	98	19.3
	Islam	7	1.4
	Christian	28	5.5
	Kirat	5	0.9
**Average family size**	4.59 + 2.19		
**Family Type**	Nuclear	281	55.3
	Joint	227	44.7
**First Pregnancy**	Yes	277	54.5
	No	231	45.5
**No. of children gave birth (N = 231)**	1	183	79.2
	2	37	16.0
	3	8	3.5
	4	1	0.4
	0 (Death / Miscarriage)	2	0.9
**Educational Background**	Illiterate	29	5.7
	Basic literate	44	8.6
	Primary School	72	14.2
	Secondary School	178	35.0
	Higher Secondary	124	24.4
	Above higher secondary	61	12.0
**Employment Status**	Self-employed / labor	16	3.1
	Business	70	13.8
	Employee	61	12.0
	Retired	4	0.8
	Unemployed	102	20.0
	House-maker	245	48.2
	Others (specify)	10	1.9
**Place of residence**	Urban	432	85.0
	Rural	76	14.9
**Experience of Child caring**	Yes	334	65.7
	No	174	34.2

### Effectiveness of pneumonia awareness program

#### Correct response from the study participants during pre and post test.

The participants who provided correct answers regarding pneumonia showed a notable increase from the pre-test to the post-test. The total correct responses in the post-test were significantly higher (P < 0.05) than those in the pre-test, signifying statistical significance in the observed improvement. This indicates a substantial positive influence of the intervention or educational program, resulting in an improved level of knowledge and understanding among the participants (Table 2).

**Table 2:** Comparison of participant's Pre-post-test response using Mc Nemar test (N = 508).

Question number	Correct Response n (%)		P-value
	Pre-test (N = 508)	Post-test (N = 508)	
Q1 (What is pneumonia?)	291 (57.3)	505 (99.4)	< 0.001*
Q2 (Who are most at risk population for pneumonia?)	299 (58.9)	440 (86.6)	
Q3.1 (Is fever a sign and symptom of Pneumonia?)	242 (47.6)	458 (90.2)	
Q3.2 (Is cough a sign and symptom of Pneumonia?)	238 (46.8)	450 (88.6)	
Q3.3 (Is chills a sign and symptom of Pneumonia?)	262 (51.6)	329 (64.87)	
Q3.4 (Is rapid breathing a sign and symptom of Pneumonia?)	216 (42.5)	404 (79.5)	
Q3.5 (Is grunting/ wheezing a sign and symptom of Pneumonia?)	161 (31.69)	323 (63.6)	
Q3.6 (Is difficult in breathing a sign and symptom of Pneumonia?)	143 (28.1)	272 (53.5)	
Q3.7 (Is vomiting a sign and symptom of Pneumonia?)	38 (7.5)	109 (21.5)	
Q3.8 (Is chest pain and chest indrawing a sign and symptom of Pneumonia?)	167 (32.9)	224 (44.0)	
Q3.9 (Is stomach pain a sign and symptom of Pneumonia?)	12 (2.4)	58 (11.4)	
Q3.10 (Is fatigue and lethargy a sign and symptom of Pneumonia?)	22 (43)	140 (27.6)	
Q3.11 (Is loss of appetite a sign and symptom of Pneumonia?)	102 (20.0)	177(34.8)	
Q4 (What is the mode of transmission of Pneumonia?)	145 (28.5)	463 (91.1)	
Q5 (Is Pneumonia transmits from pregnant mother to fetus?)	118 (23.2)	388 (76.4)	
Q6 (Can Pneumonia be prevented by vaccination?)	320 (63.)	497 (97.8)	
Q7 (Is the vaccine against childhood pneumonia available in our country?)	327 (64.4)	492 (96.8)	
Q8 (If available, how many times this vaccine needs to be given to the child?)	39 (7.7)	449 (88.3)	
Q9 (In which age, do these pneumonia vaccine need to be administered?)	76 (15.0)	396 (78.0)	
Q10 (Do we need to pay for pneumonia vaccine?)	169 (33.3)	389 (76.6)	
Q11 (Is Childhood Pneumonia a curable disease)	371 (73.0)	491(96.6)	
Q12 (What are the risk factors for childhood Pneumonia?)	79 (15.5)	308 (60.6)	
Q13 (Do exposure of second hand smoke increase the risk of Pneumonia among the children?)	247 (48.6)	487 (95.9)	
Q14 (What are the environmental risk factors for childhood Pneumonia?)	155 (30.5)	344 (67.7)	

* indicates highly statistical significant McNemar’s test value.

Calculated across all 24 individual knowledge items (508 participant * 24 items) = 12,192 total response.

Across the 24 knowledge items assessed among 508 participants (12,192 responses per assessment point), the overall proportion of correct responses increased from 34.7% (4,239/12,192) at pre-test to 70.5% (8,593/12,192) at post-test following the intervention. This corresponds to an absolute increase of 35.7% in the cumulative proportion of correct responses across all knowledge items.

#### Knowledge score among study participants in pre and post test.

The participant’s knowledge scores before and after the intervention showed significant increase of the score value from pre test (mean = 8.3, SD = 4.2) to the post test (mean = 16.9, SD = 3.4). The value of the effect size (1.6) and t test (t(507)=37.3) also indicate that the education intervention had good effect on participant’s knowledge (Table 3).

**Table 3:** Comparison of pre-post intervention knowledge scores using Paired Sample *t* test.

Variable	Pre test			Post test			Effect Size	t(df)	p-value
	Mean (SD)	Standard Error	95% CI	Mean (SD)	Standard Error	95% CI	Cohen’s d		
Knowledge score	8.3 (4.2)	0.2	7.9-8.7	16.9 (3.4)	0.15	16.6-17.2	1.6	37.3 (507)	<0.001

(SD = Standard Deviation, CI = Confidence Interval, df = Degree of freedom).

#### Domain wise change in knowledge among pregnant women due to intervention.

P-value indicates the statistical significance of the difference in means between the pre-test and post-test scores. All five domains showed a significant improvement in mean knowledge, with the highest improvement in knowledge on symptoms of pneumonia (2.8) and on vaccines (2.5), followed by risk factors (1.3), mode of transmission (1.1) and general knowledge (0.93) (Table 4).

**Table 4:** Domain wise Change in Knowledge before and after the intervention.

Domain	Pre test	Post test	Difference	P value
	Mean + SD	Mean + SD		
**General Knowledge**	1.9 + 1.0	2.8 + 0.4	0.9(0.8-1.0)	< 0.001
**Knowledge on Symptoms of Pneumonia**	2.9 + 2.4	5.8 + 2.2	2.8 (2.5-3.0)	< 0.001
**Knowledge on Mode of Transmission**	0.5 + 0.7	1.67 + 0.5	1.1 (1.08-1.2)	< 0.001
**Knowledge on Vaccine**	1.8 + 1.4	4.37 + 0.95	2.5 (2.40-2.7)	< 0.001
**Knowledge on Risk factor**	0.9 + 0.9	2.24 + 0.87	1.3 (1.2-1.4)	< 0.001

Note: Differences between pre-test and post-test mean scores were analyzed using paired t-test. A p-value < 0.05 was considered statistically significant.

The qualitative findings also indicate increased awareness and understanding among the pregnant women regarding various domains as pneumonia symptoms, vaccination schedules, risk factors and the accessibility of free immunization services which was the objective of the educational sessions. Their statements also reflect the positive impact of the awareness program on their knowledge about pneumonia and related preventive measures.

A pregnant woman who attended the educational sessions during qualitative interview demonstrated a clear comprehension of the understanding regarding the symptoms of the Pneumonia. Her ability to recall and explain these symptoms implied that the educational program was successful in raising awareness and understanding, both of which are essential for early diagnosis and treatment of childhood Pneumonia after their delivery. She articulated her understanding by stating;

*“Crackles in the chest, difficulty in breathing, fever are the symptoms of Pneumonia……….. I used to ignore rapid breathing, thinking it was normal during illness, but now I understand it can be a sign of pneumonia.”* (29 yrs pregnant woman)

The fact that the participants acknowledged that they had previously been unaware of the vaccine schedule highlights how important it is for the program to close important knowledge gaps. When asked about new knowledge they gained from the program, a woman who took the educational sessions shared her insights as;

*“Before this program, I didn't know that vaccines should be given three times”* (23 yrs pregnant woman)

*““I didn’t know vaccines could protect against pneumonia. I’ll now ensure my child gets all the recommended vaccinations.”* (30 yrs pregnant woman)

The improved knowledge of the importance and accessibility of vaccinations, as delivered by the Nepalese government, was one of the major outcomes of the educational sessions. Numerous participants said they had learned new things in this particular area. A pregnant woman, stated the following to demonstrate her heightened awareness:

*“....immunization should be done and I didn’t know it is free, now I know it is free of cost”* (27 yrs pregnant woman)

#### Change in Preferred Practice of expectant women after identification of danger sign of pneumonia.

The survey aimed to assess pregnant women's preferences in managing infections upon recognizing pneumonia warning signs. The data indicates a decrease in the percentage of respondents choosing to “wait and see” for self-recovery, dropping from 0.39% in the pre-test to 0% in the post-test, suggesting that the intervention likely enhanced awareness of the importance of seeking medical care. Similarly, fewer individuals opted for self-medication by purchasing drugs from a drugstore, declining from 7.87% in the pre-test to 2.76% in the post-test, indicating a potential shift towards seeking professional medical treatment. Notably, there was an increase from 72.83% in the pre-test to 85.04% in the post-test regarding the preference for seeking care at a medical facility as soon as possible, demonstrating the intervention's success in promoting timely medical attention. Lastly, the percentage of respondents answering “don't know” decreased from 13.98% in the pre-test to 0.98% in the post-test, suggesting that the intervention may have contributed to heightened knowledge and awareness of preferred approaches for treating illnesses (Fig 1).

**Fig 1:**
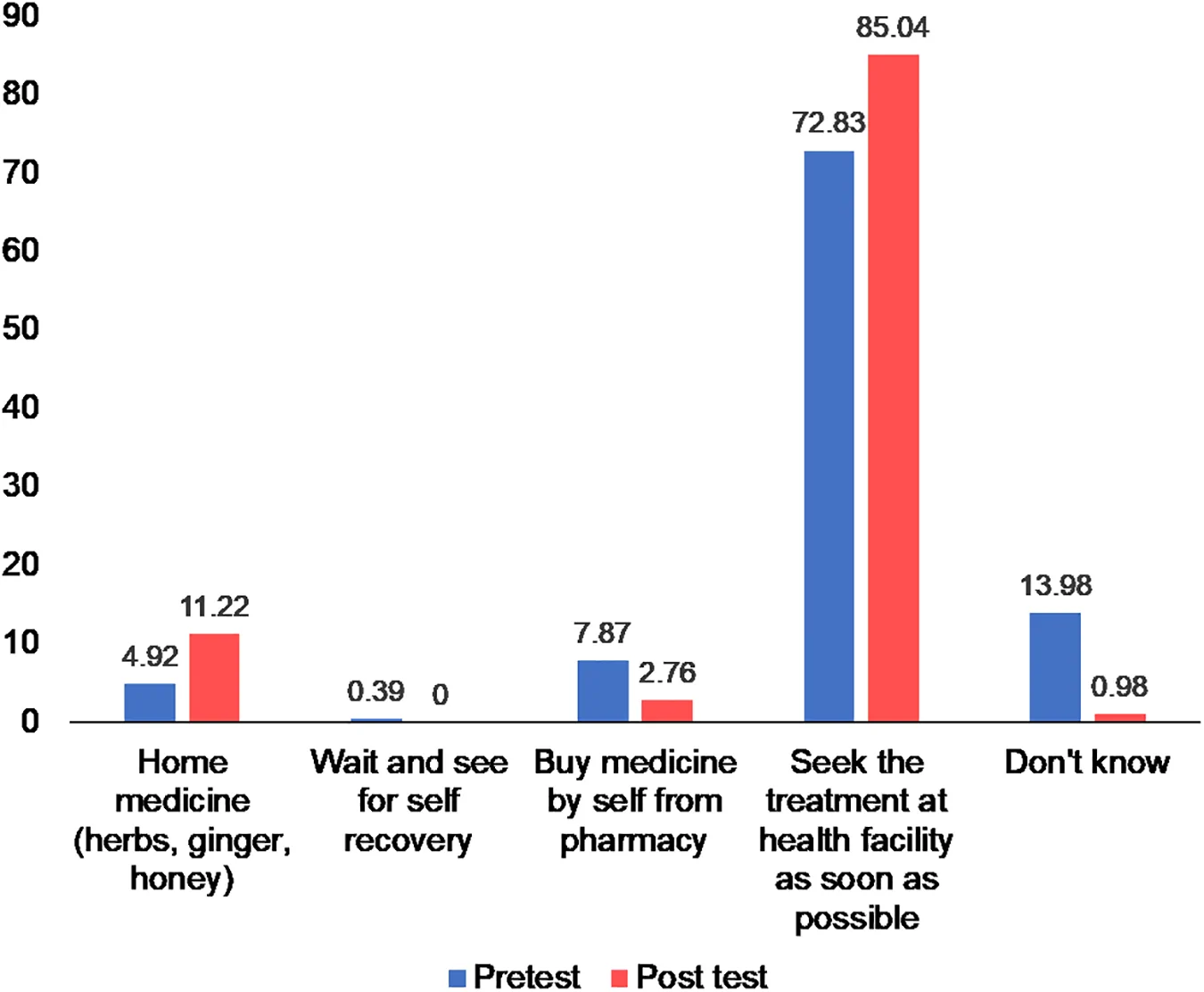
Change in Preferred practice after identification of danger sign of pneumonia.

Participants during qualitative interview were also emphasized the importance of seeking medical advice or intervention, indicating an awareness of the need for timely medical attention, especially in the presence of sign and symptoms of pneumonia.

The crucial importance of promptly seeking medical care, especially when signs of pneumonia are present, was another important topic covered in the educational sessions. In qualitative interviews, participants emphasized this point, suggesting greater awareness of the importance of seeking immediate medical attention. A pregnant woman who attended the educational program put it like way:

*“On today's program, I know that we should go for doctor as soon as possible after child get sick* ” (30 yrs old pregnant woman)

*“I now understand that fast breathing is not normal and could mean something serious like pneumonia. I will monitor it closely and act quickly.”* (27 yrs old pregnant woman)

#### Change in satisfaction with having knowledge about pneumonia.

The level of self-satisfaction with pneumonia knowledge among pregnant women changed before and after pneumonia awareness program. The majority of respondents (59.05%) were initially dissatisfied with their knowledge; but, following the program, there was a notable improvement in satisfaction, with 89.17% of respondents reporting being satisfied or extremely satisfied with their knowledge. The proportion of respondents who were extremely dissatisfied with their knowledge reduced from 23.62% in the pre-test to 1.18% in the post-test, while the proportion that were satisfied or very satisfied increased from 40.94% in the pre-test to 89.17% in the post-test (Fig 2).

**Fig 2:**
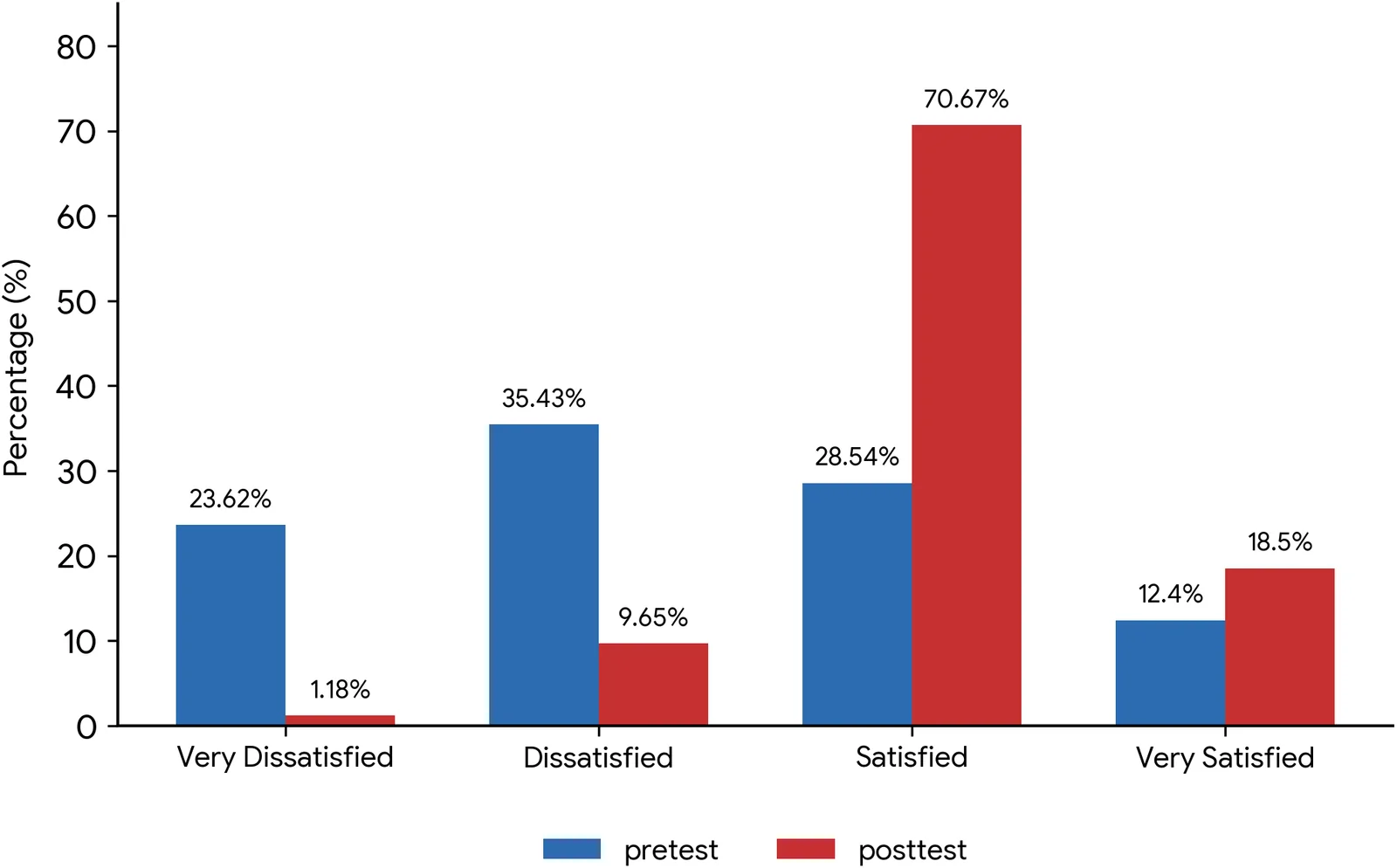
Change in satisfaction with having knowledge about Pneumonia.

The feedback received during qualitative interviews with the participating women underscores the program's effectiveness in delivering information and creating a positive impact on the attendees. These women not only value the current learning experience but also express a desire for the program to be consistently available in the future, highlighting its lasting impact and their on-going interest in its continuation.

*“I have learned and understood much more today”* (27 yrs old pregnant woman)

*“yes, the program was fruitful……. This session was very helpful in clarifying misconceptions. I also feel that I can share this information with other mothers in my community.”* (29 yrs old pregnant woman)

*“It would be great if this kind of program could be conduct repeatedly.”* (23 yrs old pregnant woman)

#### Association of Knowledge difference with socio demographic characteristics of the participants.

In the bivariate analysis assessing the relationship between knowledge difference and socio demographic variables, age, caste, total family member, education, employment and having previous experience of child caring were found to be statistically significant. However, when these variables were collectively assessed on the multivariable analysis, none exhibit a significant association (Table 5).

**Table 5:** Factors associated with change in knowledge with socio demographic traits.

Variable	Bivariate			Multivariate		
	Coefficient (95% CI)	Std Error	P value	Coefficient (95% CI)	Std Error	P value
**Age**	−0.87 (−0.18-0.00)	0.49	0.07*	−0.55 (−0.16-0.05)	0.54	0.31
**Caste**						
Brahmin / Chhetri	Ref	–	–	Ref	–	–
Madhesi	0.66 (−1.86- 3.17)	1.28	0.61	0.40 (−2.13-2.94)	1.29	0.75
Dalits	0.69 (−1.05- 2.42)	0.88	0.44	0.27 (−1.49-2.04)	0.89	0.76
Janajati	−0.75 (−1.72, 0.23)	0.50	0.13*	−0.89 (−1.89-0.99)	0.50	0.07
Muslims	1.41 (−3.21- 6.04)	2.35	0.55	0.81 (−3.94-5.58)	2.42	0.74
Others	6.21 (−4.03- 16.45	5.21	0.23	4.15 (−6.17-14.4)	5.25	0.43
**Religion**						
Hindu	Ref	–	–	–	–	–
Buddhist	0.29 (−0.87-1.45)	0.59	0.62	–	–	–
Islam	1.09 (−2.82-5.00)	1.99	0.58	–	–	–
Christian	−0.65 (−2.66-1.35)	1.02	0.51	–	–	–
Kirat	1.91 (−2.69-6.53)	2.35	0.41	–	–	–
**Total family members**	0.18 (−0.02-0.39)	0.10	0.07*	0.16 (−0.04-0.37)	0.10	0.130
**Family types**						
Nuclear	Ref	–	–	–	–	–
Joint	0.51 (−0.39-1.42)	0.46	0.27	–	–	–
**Education**						
Illiterate	Ref	–	–	Ref	–	–
Basic literate	0.32 (−2.10-2.76)	1.24	0.79	0.09 (−2.38-2.57)	1.26	0.93
Primary School	2.14 (−0.09-4.38)	1.14	0.06*	1.64 (−0.67-3.96)	1.18	0.16
Secondary School	0.25 (−2.01-2.06)	1.03	0.98	−0.42 (−2.55-1.71)	1.08	0.69
Higher Secondary	0.17 (−1.93-2.27)	1.06	0.87	−0.40 (−2.63-1.82)	1.13	0.72
Above higher secondary	0.40 (−1.89-2.70)	1.16	0.72	−0.20 (−2.60-2.20)	1.22	0.86
**Employment**						
Self-employed / labor	Ref	–	–	Ref	–	–
Business	0.70 (−2.13-3.54)	1.44	0.62	0.90 (−1.95-3.77)	1.45	0.53
Employee	0.70 (−2.17-3.58)	1.46	0.63	0.81 (−2.10-3.73)	1.48	0.58
Retired	1.68 (−4.04-7.41)	2.91	0.56	1.89 (−3.82-7.61)	2.91	0.52
Unemployed	1.63 (−1.11-4.39)	1.40	0.24	1.35 (−1.41-4.13)	1.41	0.33
House-maker	1.45 (−1.18-4.10)	1.34	0.27	1.57 (−1.08-4.2)	1.35	0.24
others	−0.21 (−4.4-3.9)	2.10	0.92	−0.46 (−4.7-3.7)	2.14	0.83
**Place of Residence**						
Urban	Ref	–	–	–	–	–
Rural	0.12 (−1.2-1.4)	0.64	0.84	–	–	–
**Experience of child caring**						
Yes	Ref	–	–	–	–	–
No	0.56 (−0.4-1.5)	0.48	0.24*	0.32 (−0.70-1.4)	0.52	0.53

*p value ≤ 0.25

Multivariable regression analysis showed that age, gender, education, and socioeconomic status were not significant predictors of knowledge improvement, indicating that the intervention effect appeared consistent across age, gender, educational, and socioeconomic groups.

#### Effectiveness of the educational intervention: Summary of qualitative findings.

***a. Increased awareness on pneumonia.***

After receiving the educational intervention the participants demonstrated an increased in their awareness of pneumonia. The participants during the interview were able to discuss pneumonia including its symptoms, mode of transmissions, risk factors and preventive measures. They acknowledged that their understanding of recognition of signs and symptoms as rapid breathing, chest indrawing and persistent cough as the critical indicators and preventive measures have been enriched due to this educational intervention. One mother after completion of the education intervention able to explain pneumonia by saying;

*“I now understand that signs like fast breathing, chest indrawing, and a high fever could mean pneumonia. I will be more alert to these symptoms in my child."* (23 yrs old pregnant woman)

Also another woman could explain about mode of transmission by stating that;

*“I thought pneumonia only spread in cold weather, but now I know it’s caused by germs that can spread through coughing and sneezing”* (29 yrs old pregnant woman)

***b. Change in health seeking behaviour.***

The participants expressed their behavioural shift towards proactive health seeking practice due to this educational intervention. Some participants with previous experience in child caring showed the change in their priority of seeking the modern health care over the traditional or home remedies. Some participant explained that they would no longer delay in seeking health care upon recognizing the symptoms of Pneumonia.

One of the women who had the previous experience of child caring expressed her shift in the behaviour by stating that

*“I used to try home remedies (as giving warm water, honey, etc.) first, but now I know the importance of going to the health center right away.”* (30 yrs old pregnant woman)

***c. Commitment to preventive practices.***

The participants in the interview displayed they were now aware of the risk factors of pneumonia due to which they showed their commitment adopting the improved hygiene, immunization and reducing the indoor air pollution. Participants shared that they now understand that the pneumonia vaccine is part of the routine immunization program. They are well-informed about the schedule, availability, and cost of the vaccine and are committed to vaccinating their children.

One of the woman at her first pregnancy shared that

*“I had never heard of pneumonia vaccines before. I’m going to ask the doctor about it during my next visit. Now that I understand their importance, I’ll ensure my child receives them on schedule.”* (23 yrs old pregnant woman)

***d. Perceived effectiveness of the program/satisfaction.***

When asked about their perceived satisfaction or effectiveness of the program, all most all participants described it as highly effective in increasing their awareness level and expressed gratitude to the intervention organizers. Some also explained that this learning will extend beyond them, as they could share this knowledge to their families and relatives. Some participants suggested that similar programs should be conducted on a regular basis. The participants found that audiovisual aids very appealing and liked the brochure and leaflets distributed, saying that they would be useful in their future use for knowledge retention. A woman express her perception regarding program by saying:

*“The session was very informative, and the visuals made it easy to understand, even for someone like me who hasn’t studied much.”* (30 yrs old pregnant woman)

*“The posters and hand-outs to take home are very helpful; they will remind me of what I learned today and allow me to share the information with others.* (23 yrs old pregnant woman)

*“The session helped me realize that even a simple cold, if ignored, can lead to something serious like pneumonia."* (27 yrs old pregnant woman)

***e. Recommendation from the participants.***

Participants were asked for suggestions to improve the program. Most of them said similar programs should be conducted in the future. Some suggested including fathers or other male family members in these sessions so the knowledge wouldn’t be limited to mothers. They felt this would make the learning more effective since men also influence healthcare decisions. For example;

*"The session was informative, but I think including fathers and grandparents would make it more effective since they also influence decisions about health care."* (27 yrs old pregnant woman)

Another woman in the interview suggested that;

*“It would be helpful if such sessions were organized regularly and covered other common diseases too.”* (23 yrs old pregnant woman)

#### Triangulation of the quantitative and qualitative findings.

***Area of convergence.***

The quantitative findings demonstrated an increase in participants’ knowledge following the educational intervention. Significant improvements were observed across all five domains: general knowledge about pneumonia, awareness of its symptoms, understanding of its mode of transmission, knowledge about vaccines, and recognition of risk factors. Similarly, the qualitative study revealed that participants perceived a transformation in their understanding of these areas. They shared how the intervention clarified previous misconceptions they had about pneumonia, leading to a better comprehension of the disease.

The findings regarding the change in self-satisfaction in having knowledge about pneumonia between quantitative and qualitative findings also aligns with each other. Both showed an increase in satisfaction among pregnant women after participating in the educational intervention.

***Area of divergence.***

No significant area of divergence was noted between quantitative and qualitative findings.

## Discussion

As being a primary caregiver of the infants and children, the maternal awareness on the childhood pneumonia is very important. An aware mother can involve in identifying the danger signs required for the early detection and prompt treatment, ensure the complete immunization and practice the good hygiene, which obviously leads to better health outcome of the children [50]. The Health Belief Model explains that, the individuals who perceive the severity of an illness and believe in the benefit of taking specific actions to reduce the risk of the illness are more likely to adopt the health promoting behaviours [51].This could be conceptualised to illustrate how mothers could be motivated to take preventive actions, such as adopting preventive behaviours as vaccination, reducing risk factors. This study showed the low level of knowledge score during the pre-test session indicating the poor knowledge among expectant mothers attending at urban hospital. The findings align with the Indonesian study, which also showed very low knowledge among the mothers in the urban community visiting the public hospital [52]. Also another community based study conducted at the Portugal explained the greater need of health education session regarding respiratory illness among the mothers or caregivers in the both intervention and control group [53]. Lack of access to good health information and a particular health education program on pneumonia in urban areas could be the reason for this. Furthermore, the busy urban lifestyle may cause people to place more importance on immediate financial issues than on health education. These challenges might be different for those in the rural areas, thus the contextualization of this intervention in the rural context as mobilization of community health volunteers, use of local relevant examples and language considering their cultural values is essential for feasibility and effectiveness. According to the survey post-test findings compared to the pre-test findings, pregnant women had a much higher score on knowledge of pneumonia after the program. Statistics showed that there was a statistically significant difference in the mean scores between the pre-test and post-test responses. This result is comparable to other studies’ findings that, following a health education program, almost all mothers could give accurate answers to questions about pneumonia [54]. Another study revealed that mothers’ knowledge of acute respiratory infection increased from 25.6% to 69.8% as a result of health education interventions [55]. In the same vein, the studies conducted in diverse geographic settings as in Bangladesh, India, Egypt, Portugal also reported a significant improvement in the knowledge regarding pneumonia among the mothers [25,53,56,57]. The similarities obtained in the findings in these studies can be due to structured and focused approaches of the health education program which effectively enhance the knowledge and awareness among the participants. The significant improvement in the mother’s knowledge in the current study was probably because of the audio-visual aids used for better learning, which is supported by the Duel Coding Theory [58]. This theory explains that the teaching method using both words and images will help for effective learning. Thus, in this study, the intervention utilized multiple materials and methods to reinforce learning. Educational brochures provided participants with a tangible reference that they could revisit after the sessions, supporting recall of key messages. Videos offered visual and auditory demonstration of health practices, making abstract concepts easier to understand. Group discussions allowed participants to ask questions, share experiences, and clarify misconceptions, which enhanced understanding and retention. The combination of these approaches contributed to the observed improvement in knowledge scores.

The present study showed that knowledge about sign and symptoms of pneumonia and vaccines of participating pregnant women was significantly improved, followed by the risk factors, mode of transmission and overview of pneumonia. The findings of this study are congruent with another study from conducted among mothers in Egypt, which found that their knowledge about the signs and symptoms of pneumonia increased from approximately one-third to two-thirds following the implementation of an educational program [26]. Moreover, the study conducted in Peru also reported that mothers who had heard about pneumonia educational campaign through television had more knowledge on identifying the signs of pneumonia than other mothers [24]. Another study conducted in Portugal also demonstrated that the group of women who participated in the health education sessions provided comparatively high numbers of the correct answers regarding the identification of the signs and symptoms [53]. The structured program and media campaigns were designed to provide clear, accessible and relevant information, which the participants can readily comprehend and remember. The application of the principles of adult learning [59], such as the need for practical and pertinent information, are likely adhered to in these programs, leading to significant improvements in knowledge. Adult learners are more likely to engage with and retain information that directly addresses their current needs or concerns.

The survey also aimed to assess preferred practices for treating illnesses after identifying danger signs of pneumonia. The result of the study showed a decrease in the number of individuals who preferred to “wait and see” or self-medicate, and an increase in those who sought treatment from a health facility. About 89.17% of respondents reported being satisfied or very satisfied with their knowledge of pneumonia after the program, compared to just 40.94% in the pre-test, indicating a considerable improvement in self-satisfaction with knowledge of pneumonia. The findings of the study is are line with another study in which mother’s knowledge and practice improved after being exposed to health education media [56]. Likewise, another study also showed that providing health education is an effective way to improve mothers’ understanding and encourage them to change their behaviour to prevent pneumonia [55]. Furthermore, a community-based randomized controlled trial conducted in Nepal found that an intervention incorporating health education for mothers resulted in increased health post visits for children under five with severe ARI [29]. This outcome can be attributed to the mothers’ enhanced ability to recognize danger signs and symptoms, leading to improved health-seeking behavior. The changes in knowledge and practices among participants can be explained by the way the educational program was designed and delivered in a hospital setting. The structured health education program employed clear and relevant materials, adhering to adult learning principles by emphasising practical and actionable knowledge. Interactive sessions, visual aids, and educational materials to take home reinforced learning and helped participants retain information. The mechanisms of change observed in this study can also be linked to key constructs of the behavioural theories, including increased perceived susceptibility and severity after understanding the risks of pneumonia, coupled with cues to action provided during the intervention. These elements likely motivated participants to absorb and retain information more effectively. Conducting the intervention in a hospital setting created a comfortable and familiar environment for the participants, making it easier for them to ask questions and engage in discussions. The educational content was aligned with cultural norms and tailored to address common misconceptions and knowledge gaps, particularly about recognizing danger signs, how pneumonia spreads, and the importance of vaccination. Firstly, it was in the Nepali language with simple words understandable to all the participants and incorporated the local common relevant examples. The common real life examples with local practices and myths were well discussed which provided the clarification towards their knowledge. The sessions also considered the gender and family dynamics to ensure the cultural appropriateness. Required adjustments were made during the pretesting phase based on the feedbacks obtained from the participants to enhance the clarity and relevance. This approach empowered participants to make informed decisions about their health and seek timely medical care when needed.

The study effectively demonstrates the Pneumonia awareness program’s success in significantly improving pregnant women’s knowledge; however, some limitations should be noted. First, the reliance on self-reported data introduces the possibility of response bias. Participants may have overstated their knowledge or changes in behaviour due to social desirability. This could lead to an overestimation of the program’s effectiveness. Although the intervention significantly increased participants’ knowledge, the study did not include follow-up assessments to evaluate whether these knowledge gains translated into sustained health-seeking behaviours. Future studies should include longitudinal follow-up to assess the impact of increased knowledge on actual behavior over time. Since being a quasi-experimental pre-post design, there is lack of a control group that limits the ability to establish causality between the intervention and the observed outcomes. Also, the sample size was calculated only for the primary outcome (change in knowledge level), and separate calculations for secondary or explanatory variables were not performed. Moreover, this design does not control for all confounding variables, such as participants’ socioeconomic background, access to healthcare, or prior exposure to health education. These uncontrolled variables might have influenced the outcomes, making it difficult to isolate the effects of the intervention. The study was conducted in a hospital setting and involved pregnant women, primarily from urban areas. This over representation of participants from urban areas limits the generalizability of the findings to rural settings or other population groups. The intervention's effectiveness might vary across different socioeconomic and cultural contexts.

## Conclusion

The study demonstrated significant improvements in participants’ knowledge and practices related to childhood pneumonia following the educational intervention. Statistically significant improvements were observed across all domains, including knowledge of pneumonia symptoms, modes of transmission, vaccination, and risk factors (p < 0.05). Statistically significant changes were observed across all domains, and health-seeking behaviours also improved, with more participants opting to seek treatment at health facilities rather than self-medicating or waiting. Additionally, the majority of participants reported increased satisfaction with their knowledge post-intervention, highlighting the effectiveness of the program. To foster the well-being of both mother and child, similar interventions or awareness programs should be conducted regularly. Health education serves as a crucial instrument for instigating behavioural changes, as it enhances knowledge and significantly impacts a mother's actions to prevent childhood pneumonia. In terms of sustainability, scaling up the program will require ongoing logistical, financial, and community support. To ensure continued success, partnerships with local health authorities, integration into routine healthcare services, and potential funding sources for long-term implementation should be explored. Additionally, ongoing training for healthcare providers and community engagement will be critical to maintaining the program’s effectiveness.

### Recommendation

To improve future interventions and research, several recommendations can be made. Incorporating a randomized controlled trial (RCT) design with a control group would provide stronger evidence of causality and enhance the reliability of findings. Combining self-reported data with objective measures, such as healthcare records or vaccination rates, could reduce biases and validate the results. Expanding similar interventions to rural and community-based settings, with tailored content for diverse demographics, would improve the program’s reach and impact. Including family members, such as fathers and grandparents, in the intervention could foster a more holistic approach to health education. For scalability, digital platforms and mobile applications could be utilized to disseminate information cost-effectively. Additionally, integrating such interventions into routine maternal and child health services and conducting follow-up sessions would ensure sustainability and long-term impact.

## Supporting information

S1 File Educational materials. (PDF)

S2 File Data collection tool. (PDF)
