# Supplementary material for: Effectiveness of childhood pneumonia awareness program among pregnant women: A hospital based quasi experimental study
Source: PLoS One. 2026 Nov 6;21(11):e0342216. doi: 10.1371/journal.pone.0342216 (PMC13742872; doi:10.1371/journal.pone.0342216)
Supplement: Supplementary file 1 [file pone.0342216.s001.pdf]

निमोनिया के कारणले हुन्छ ?

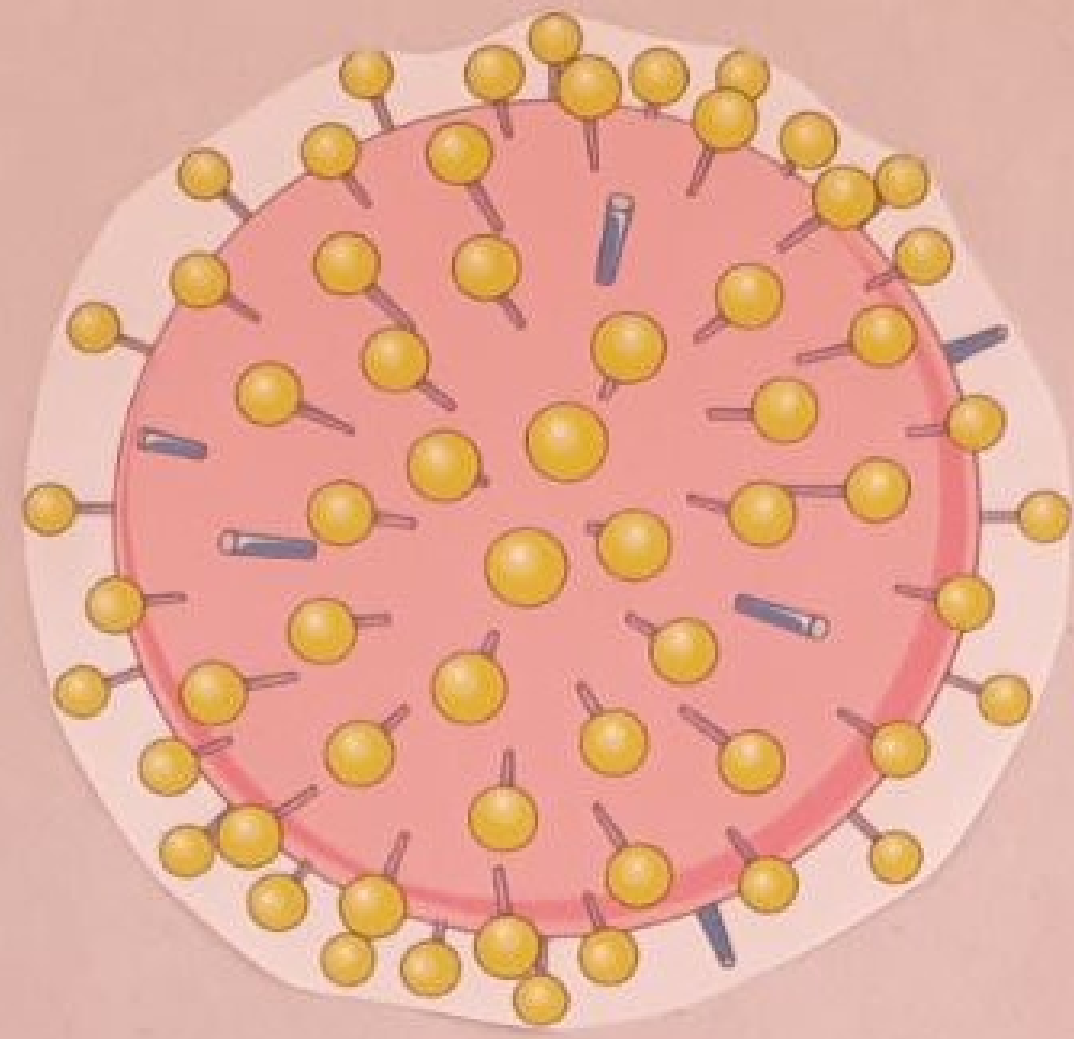

भाईरस

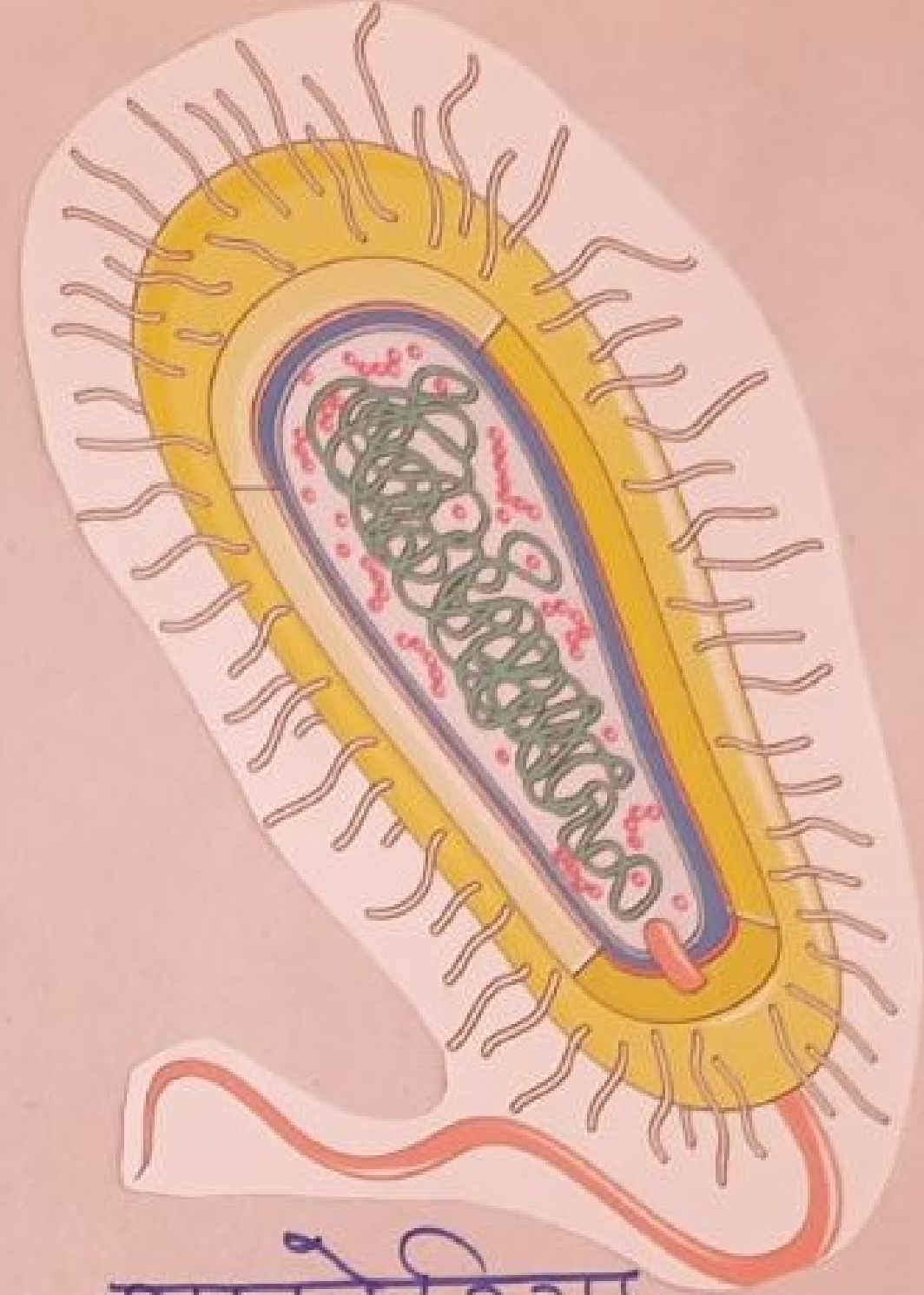

ब्याक्टेरिया

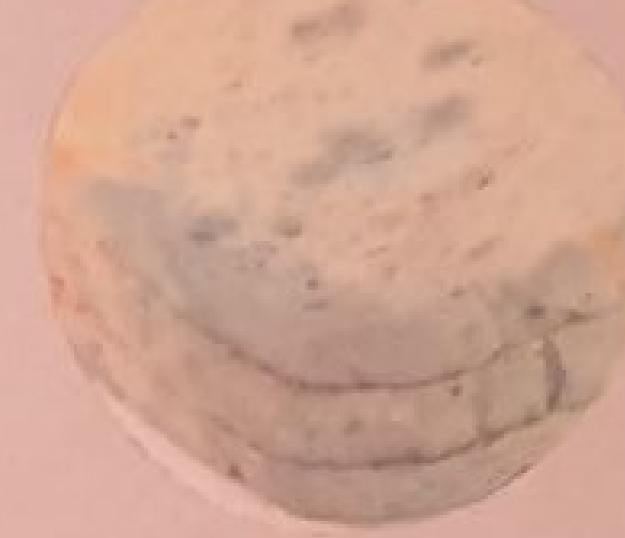

ढुसी

संक्रमित व्यक्तिले/को/सँग

- हाच्छ्यु गर्दा
- खोक्दा
- थुकको छिटा पर्दा
- हात मिलाउँदा

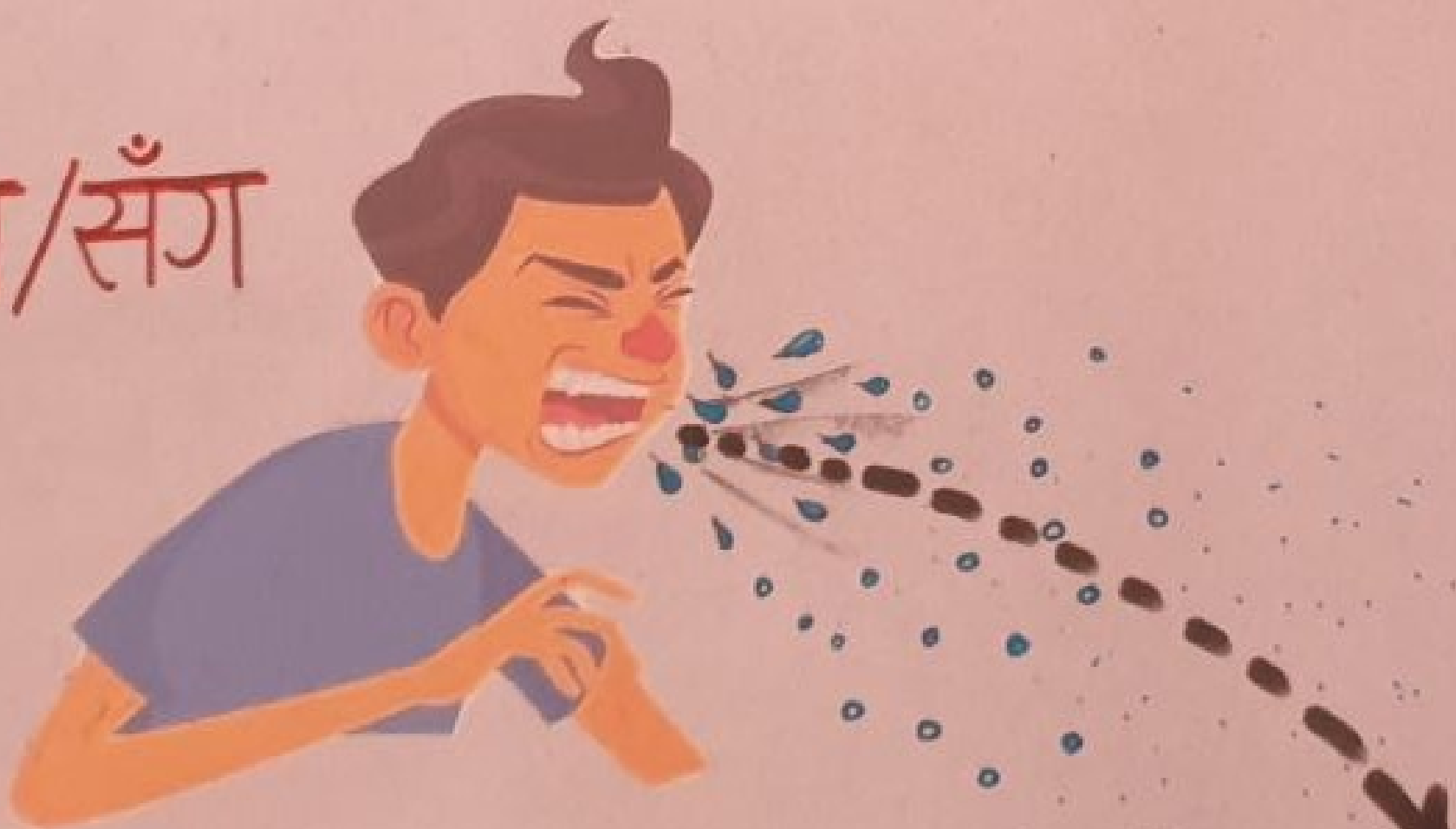

निमोनिया कसरी सर्दछ ?

बढी जोखिम कसलाई हुन्छ ?

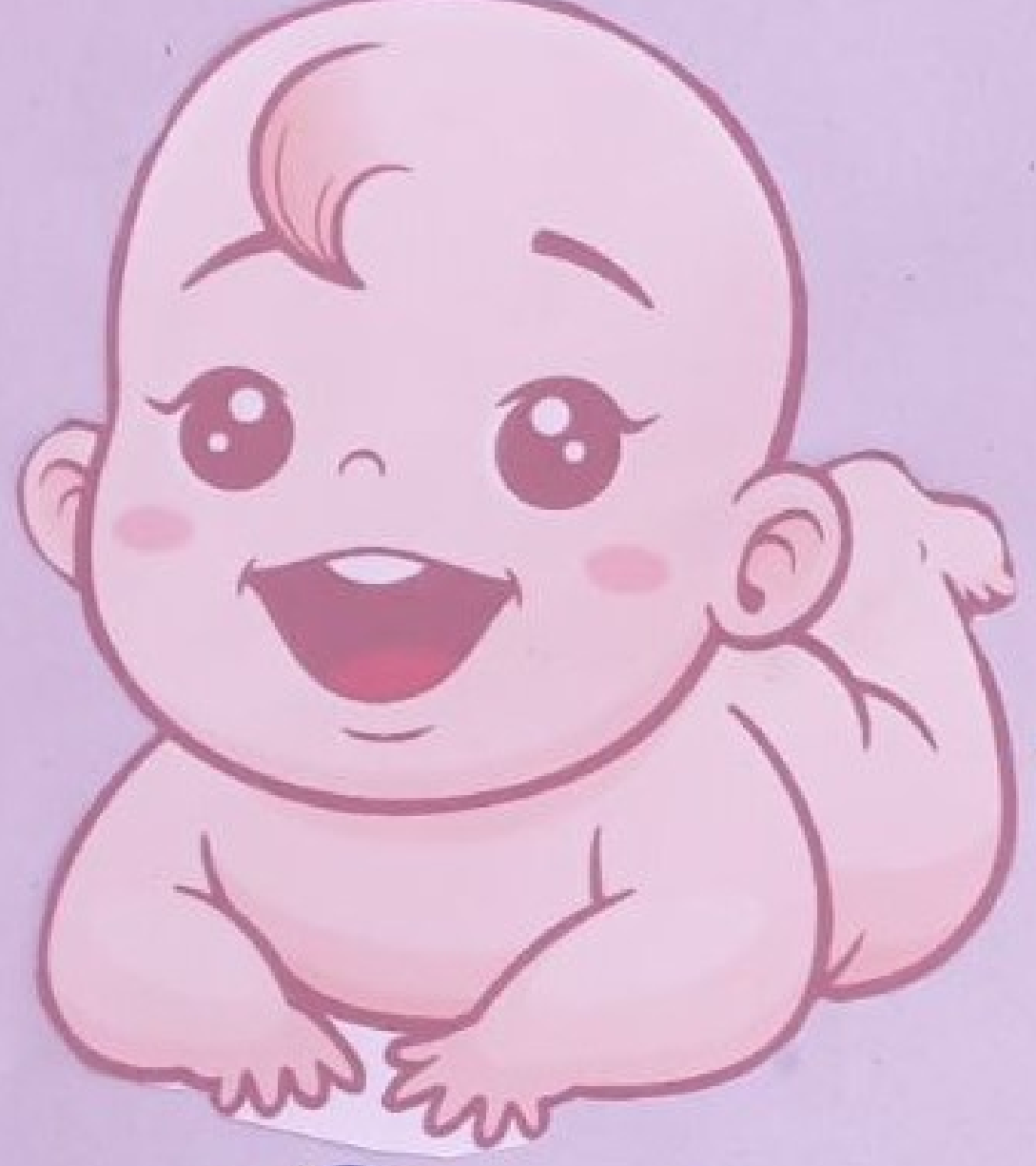

५ वर्ष मुनीका बालबालिका

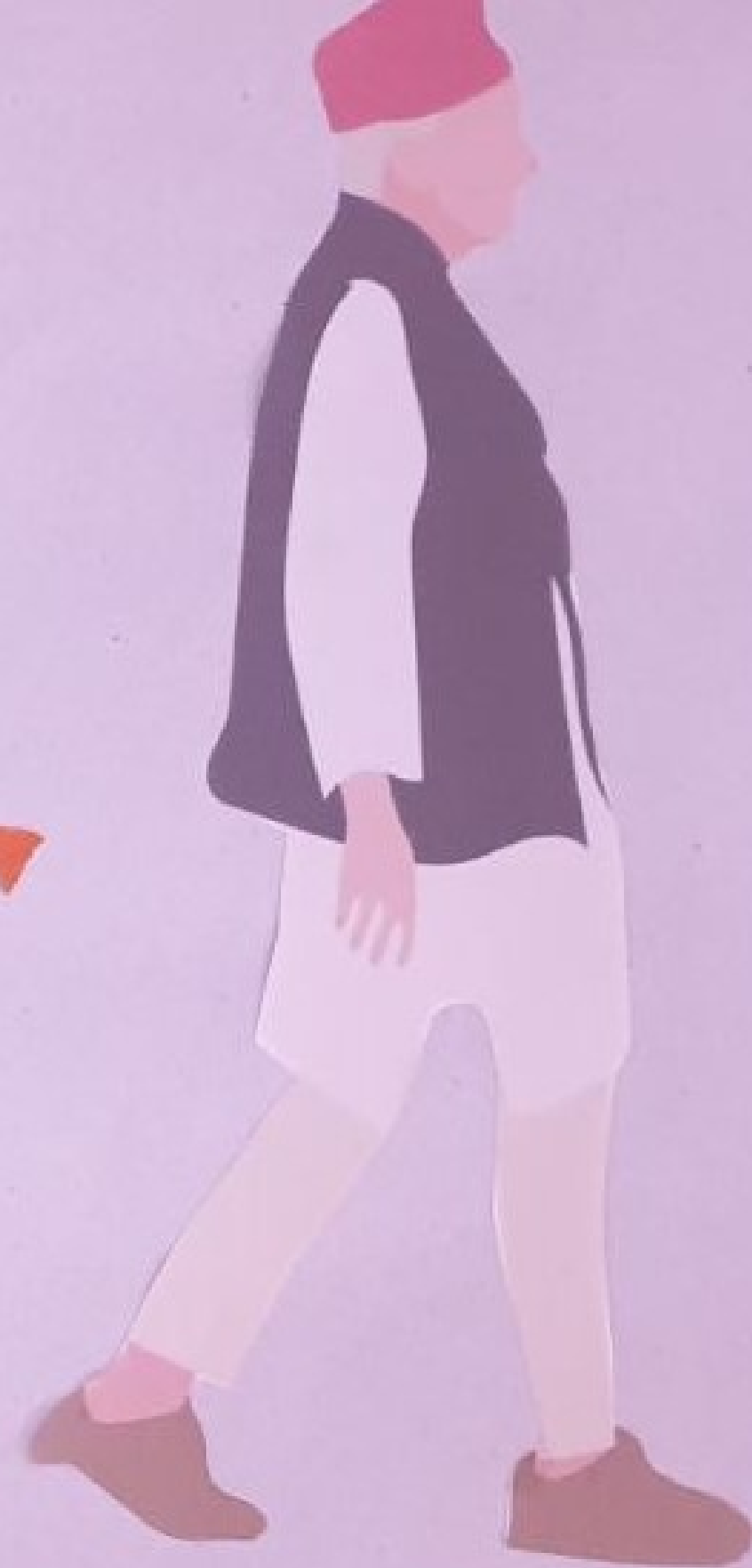

वृद्ध-वृद्धा

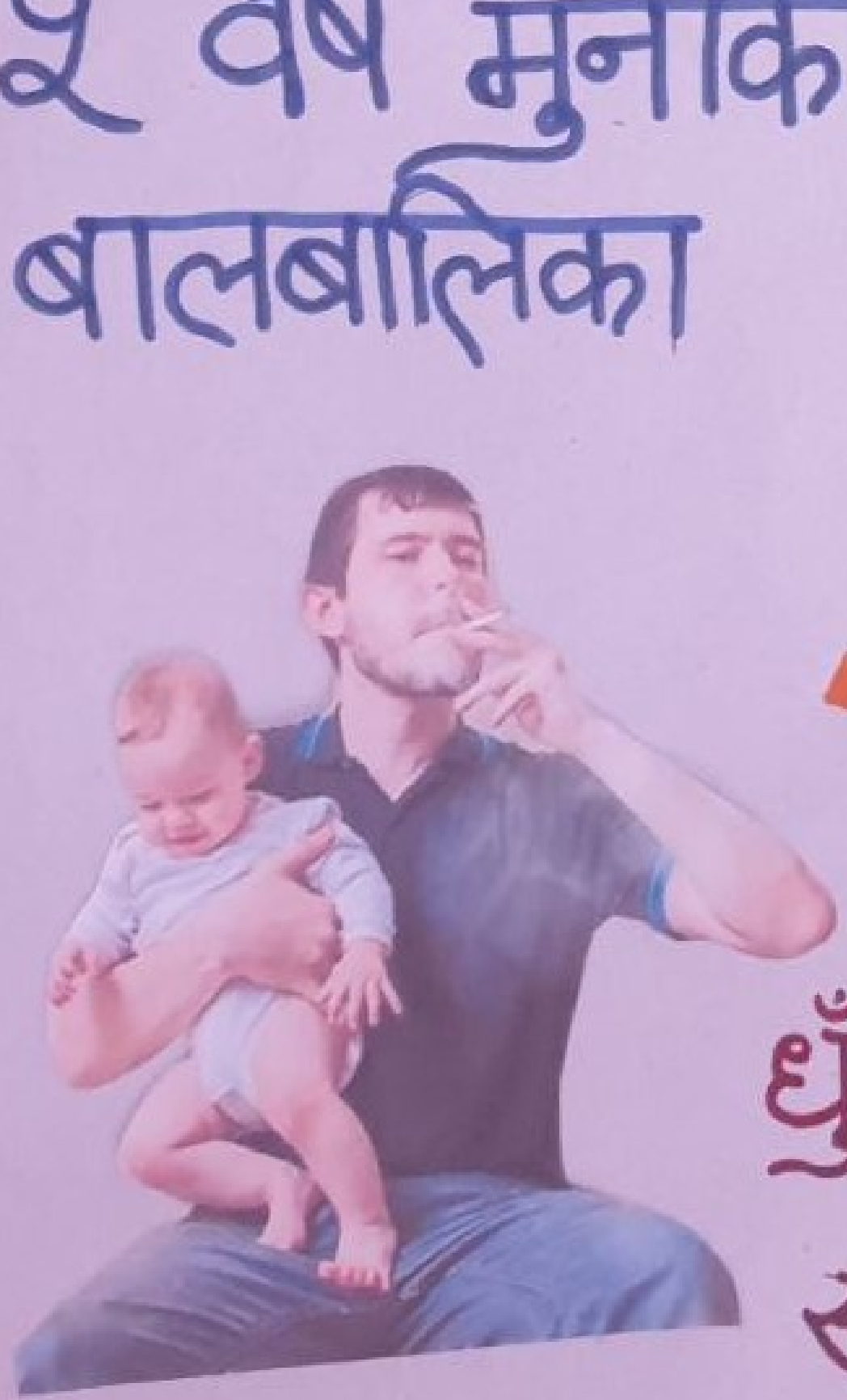

धुँवा धुलोको बढी सम्पर्कमा रहेका

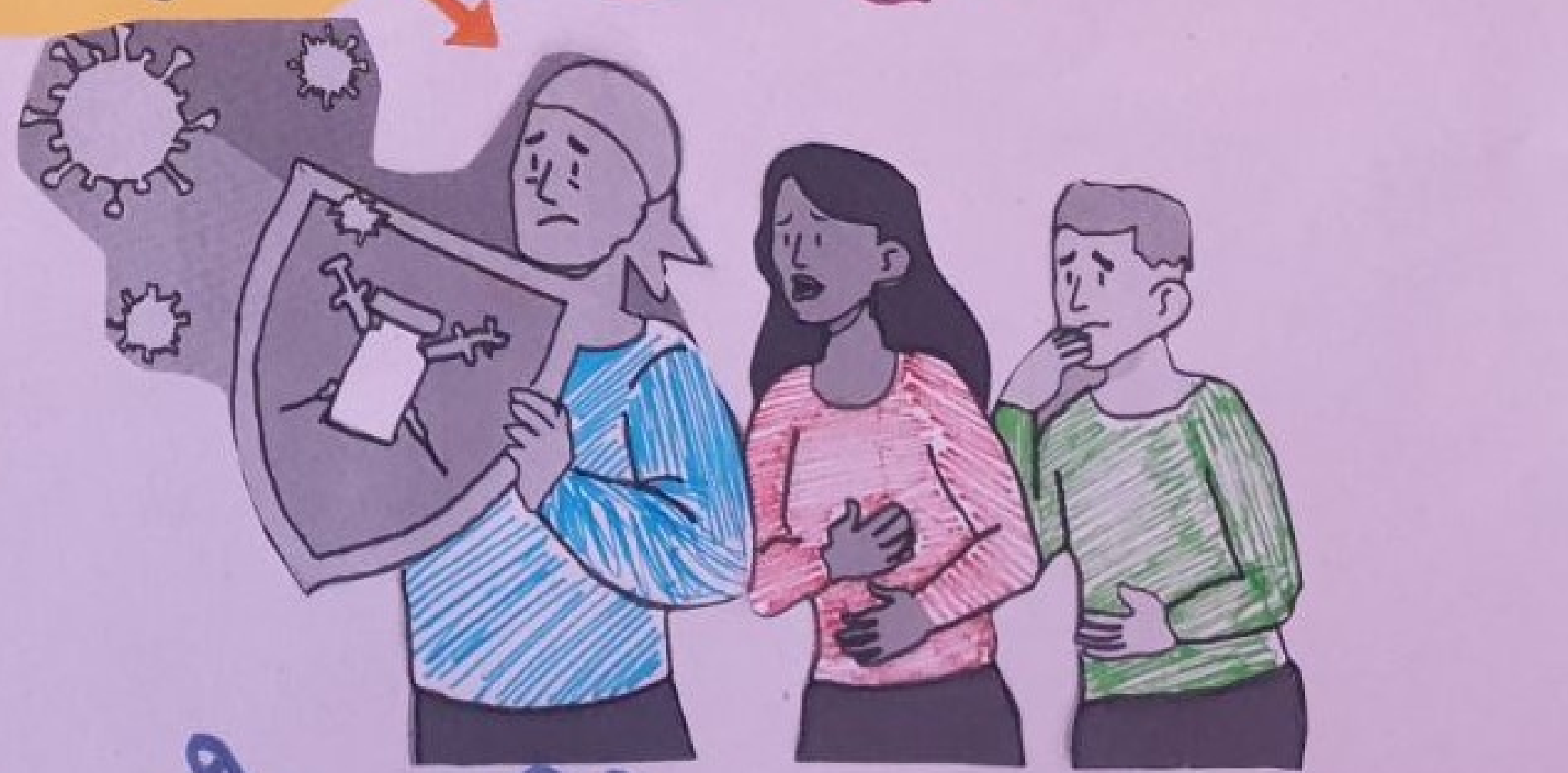

रोग प्रतिरोधात्मक क्षमता कम भएका

निमोनियाको रोकथाम र उपचार

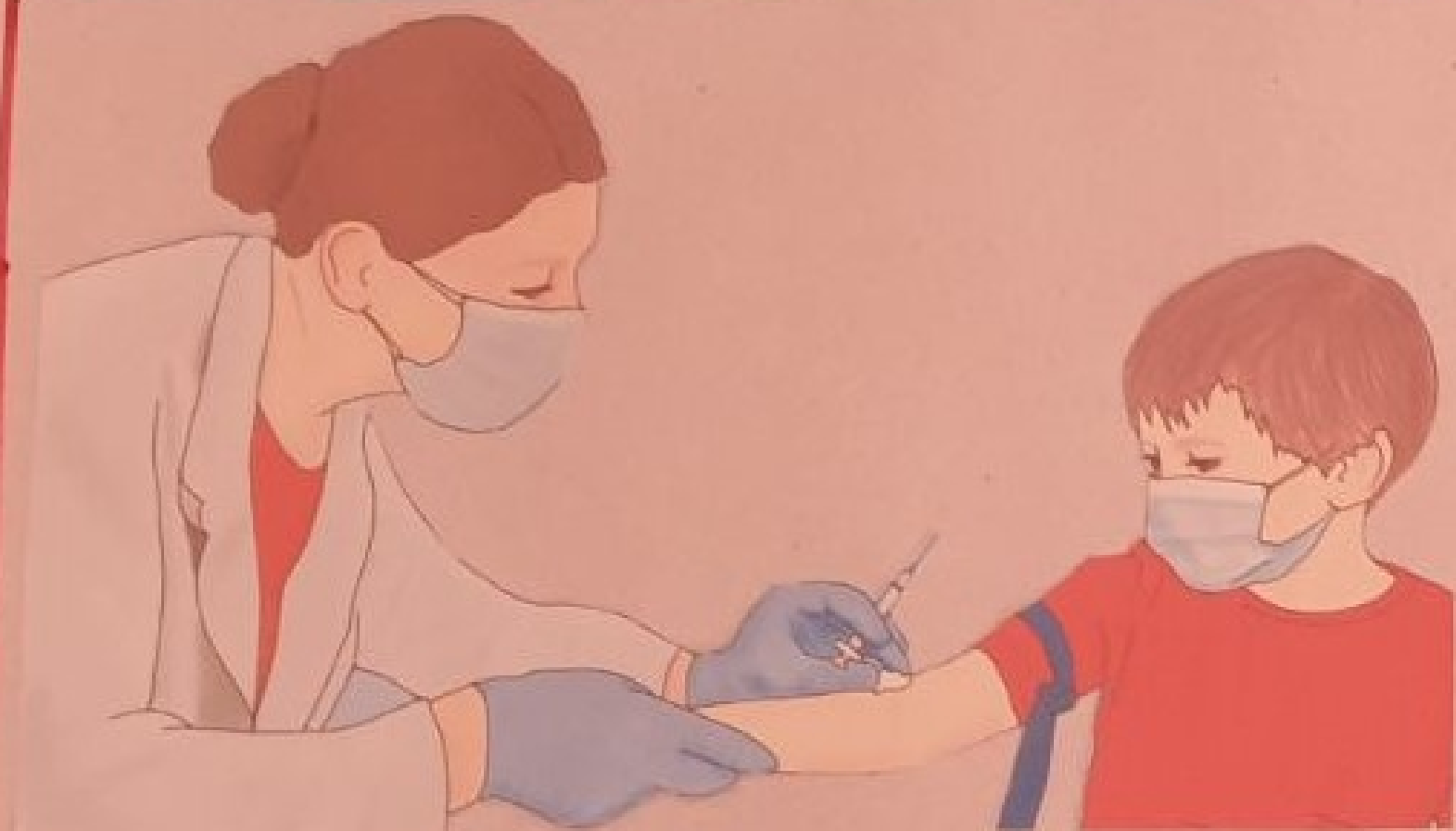

i. समयमै पुरै खोप लिने

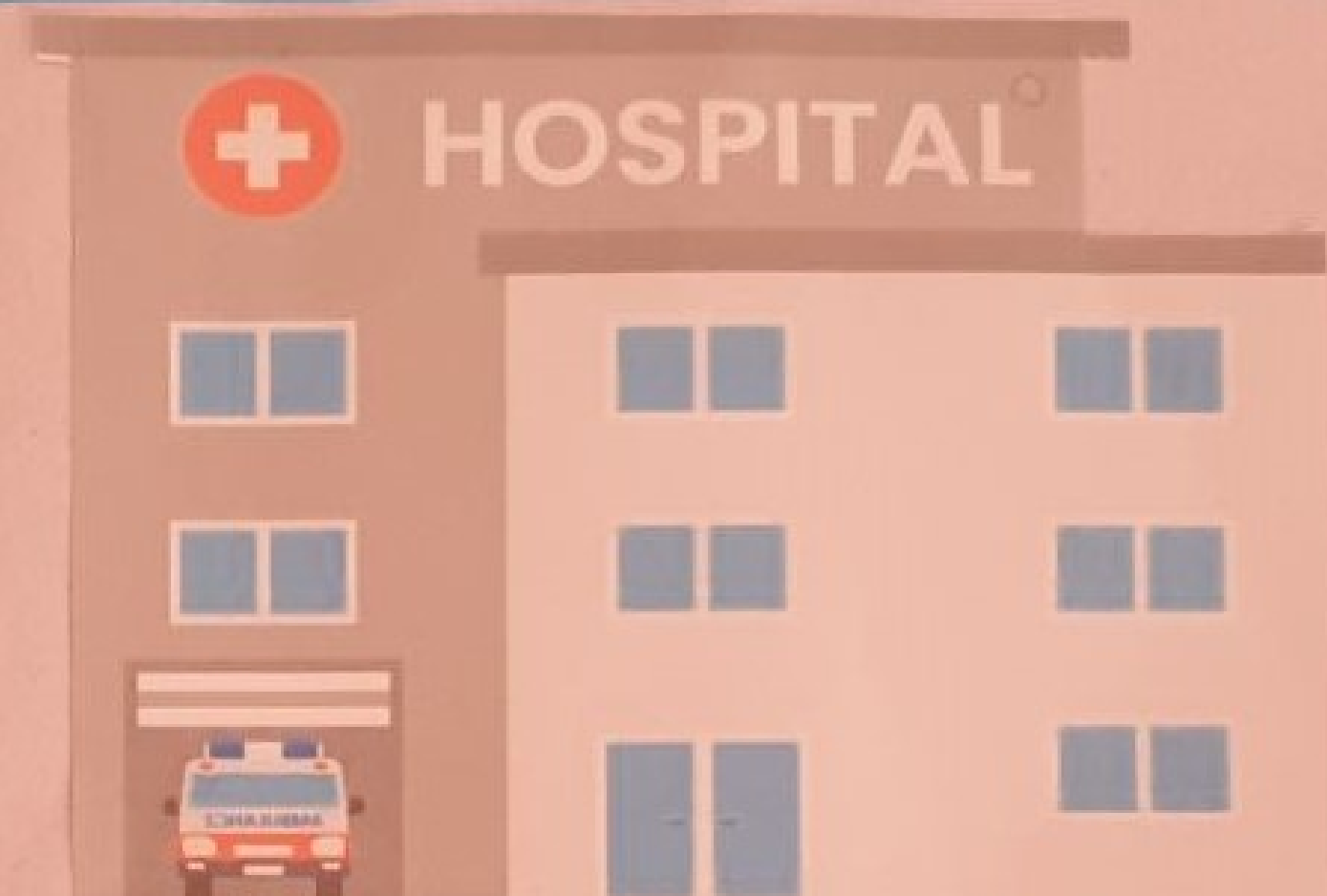

ii. गाह्रो भए तुरुन्तै अस्पताल जाने

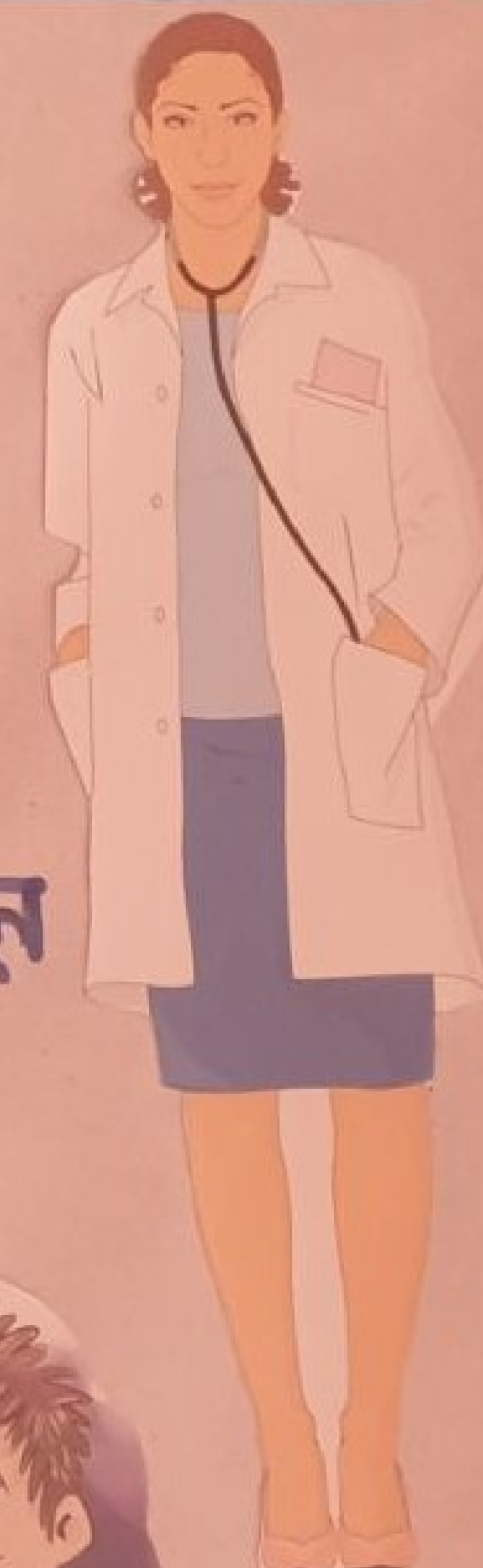

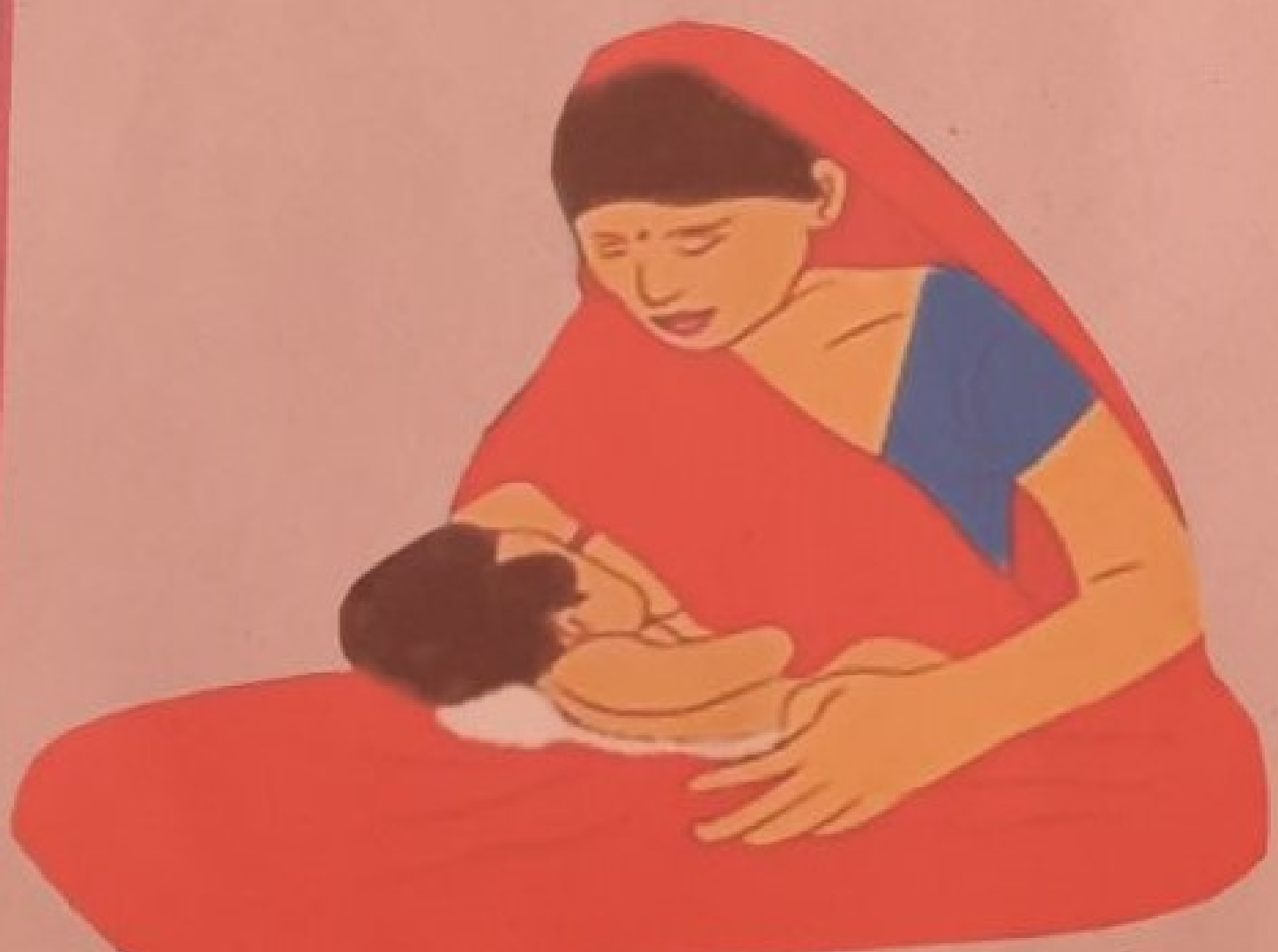

iii. पूर्ण स्तनपान गर्ने

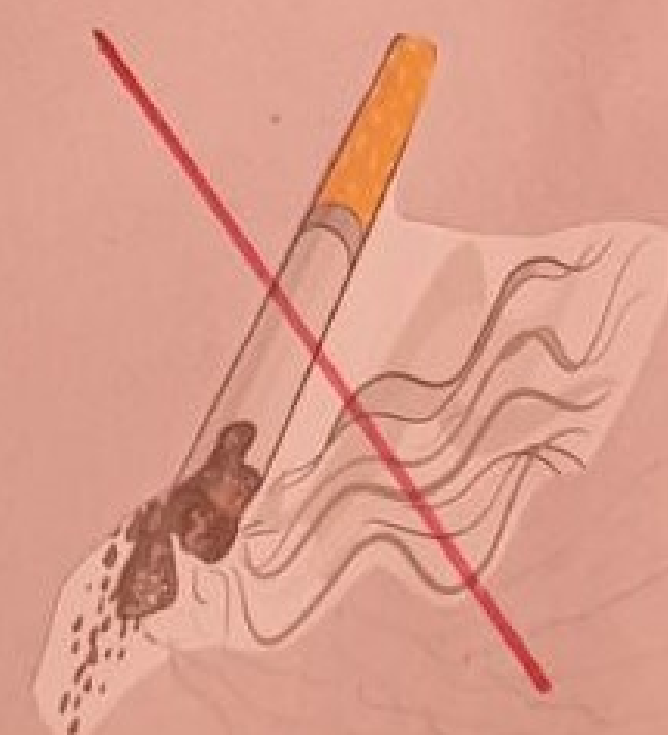

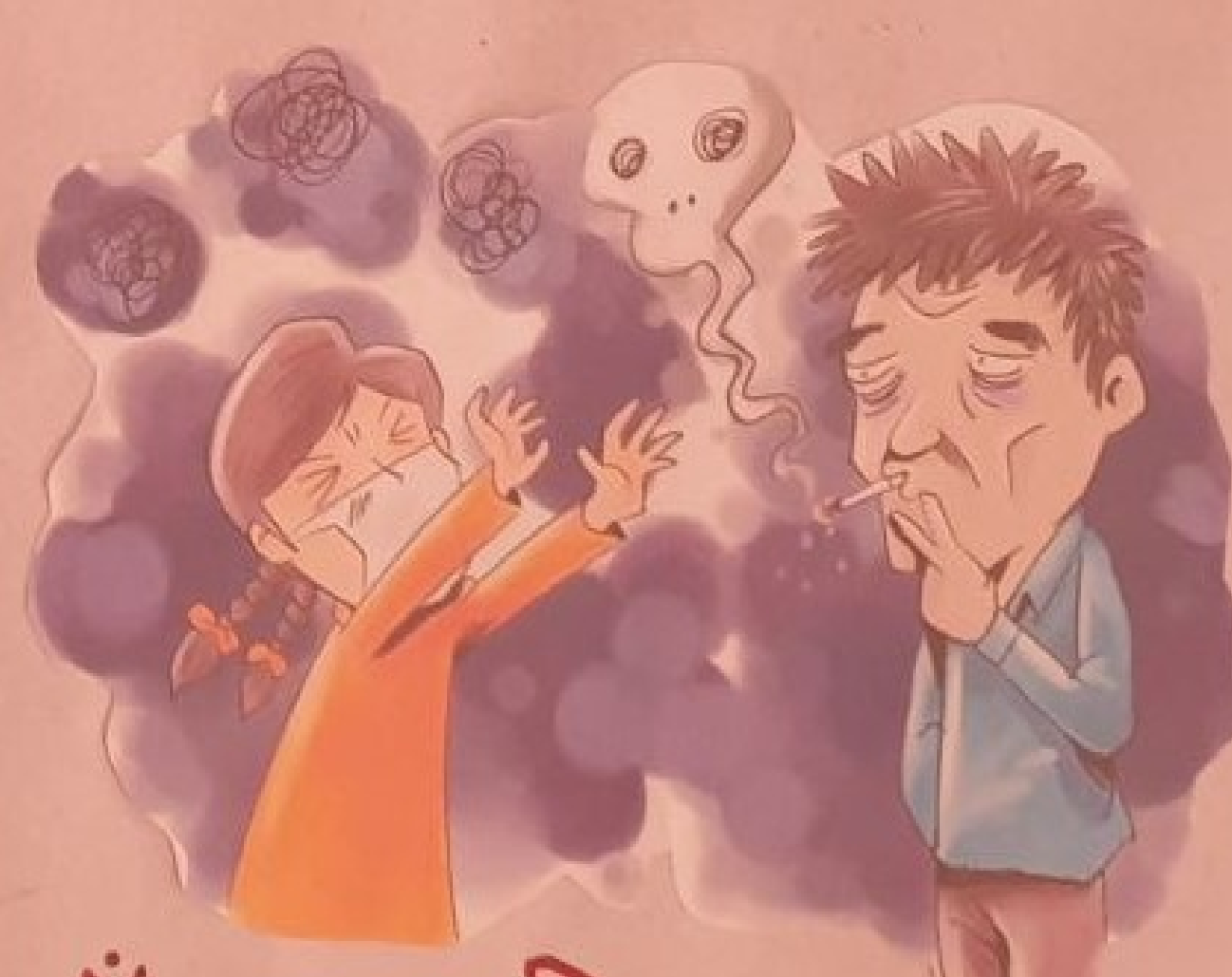

iv. धुँवा धुलोबाट बचाउने

स्वस्थ फोक्सो

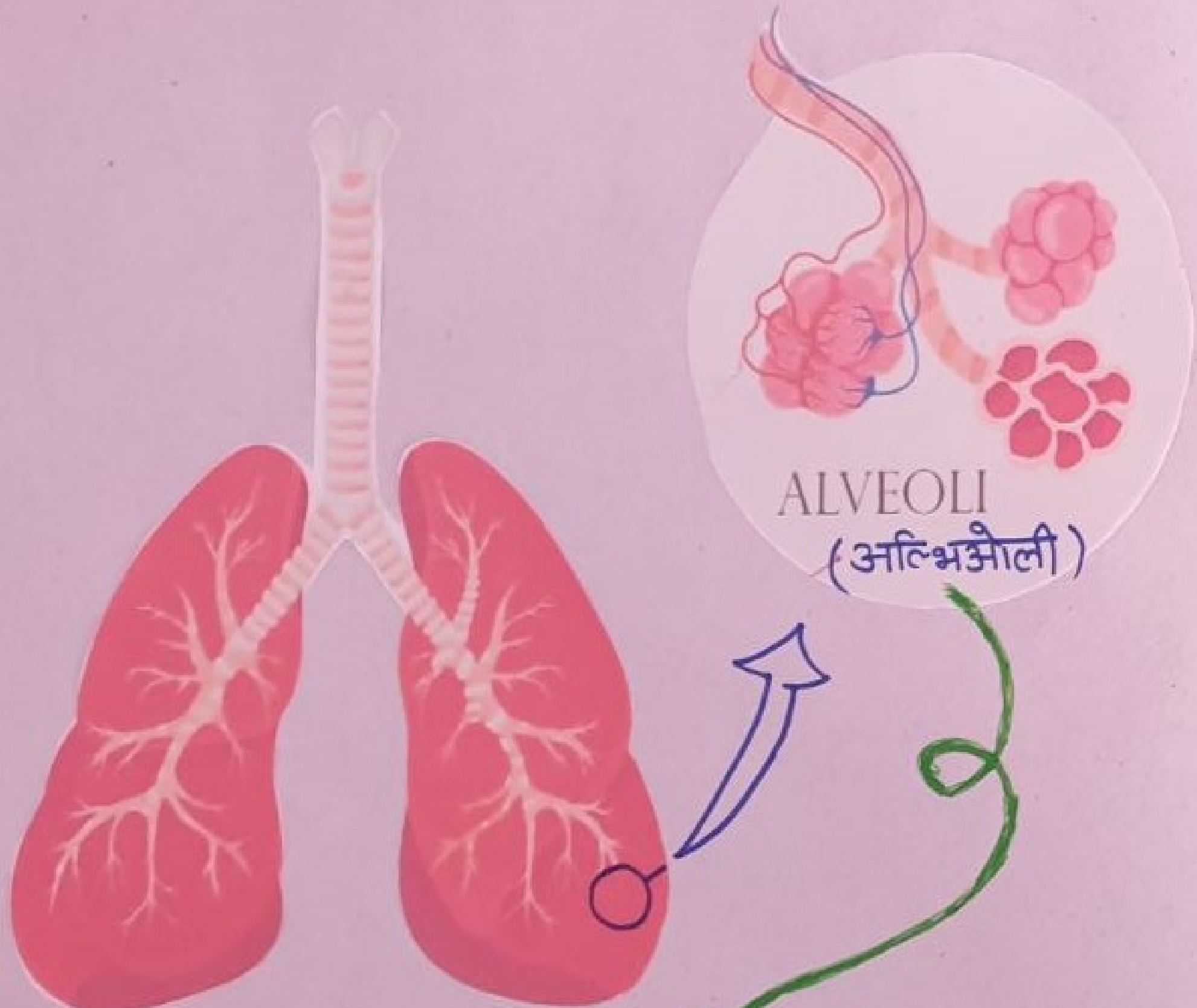

फोक्सो भित्र भएका
स-साना
हावाका थैलीहरू

निमोनिया संक्रमित फोक्सो

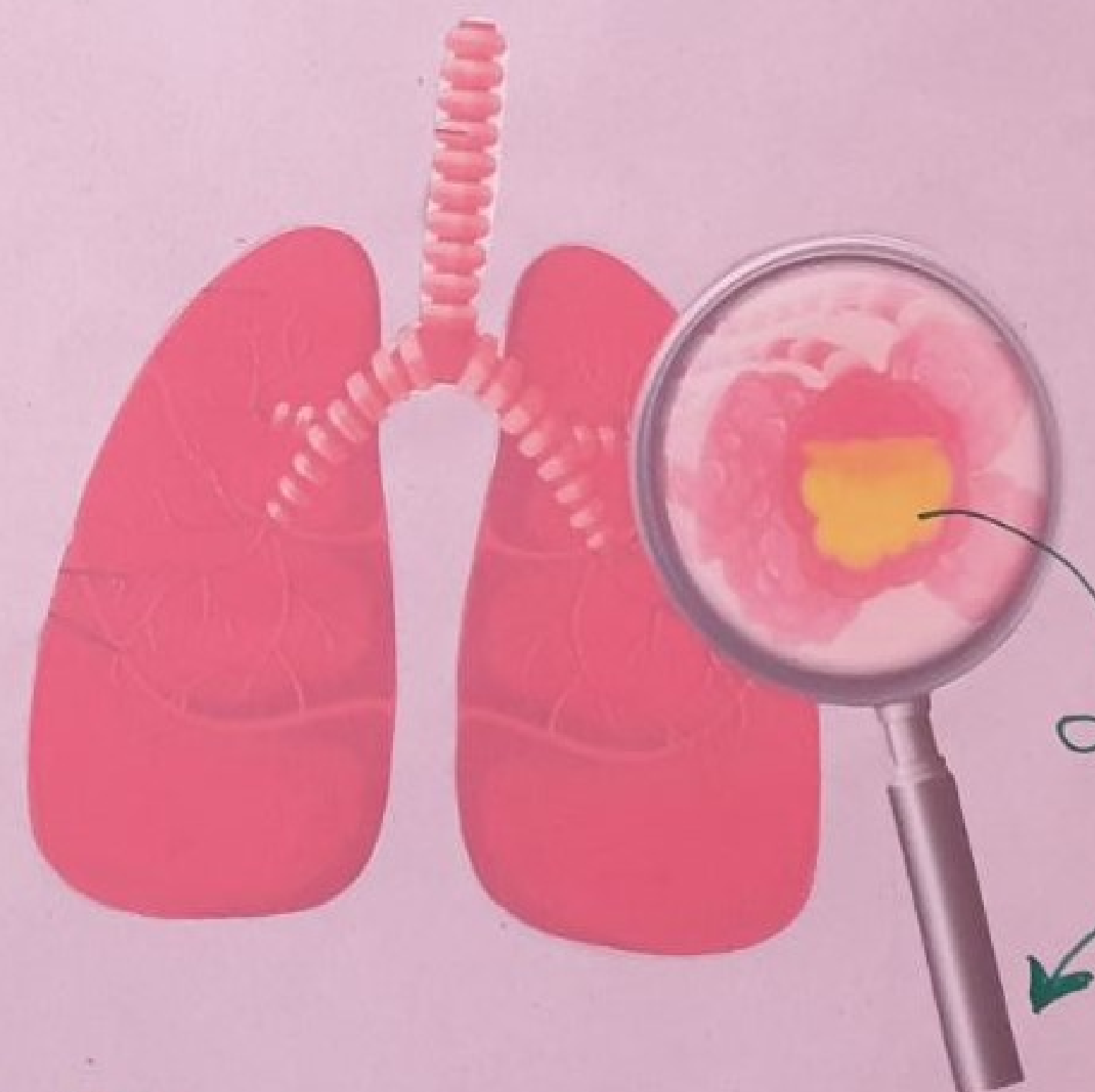

निमोनिय भएपछि स-साना
हावाका थैलीहरूमा(अल्भिओली)
तरल पदार्थ भरिन्छ।

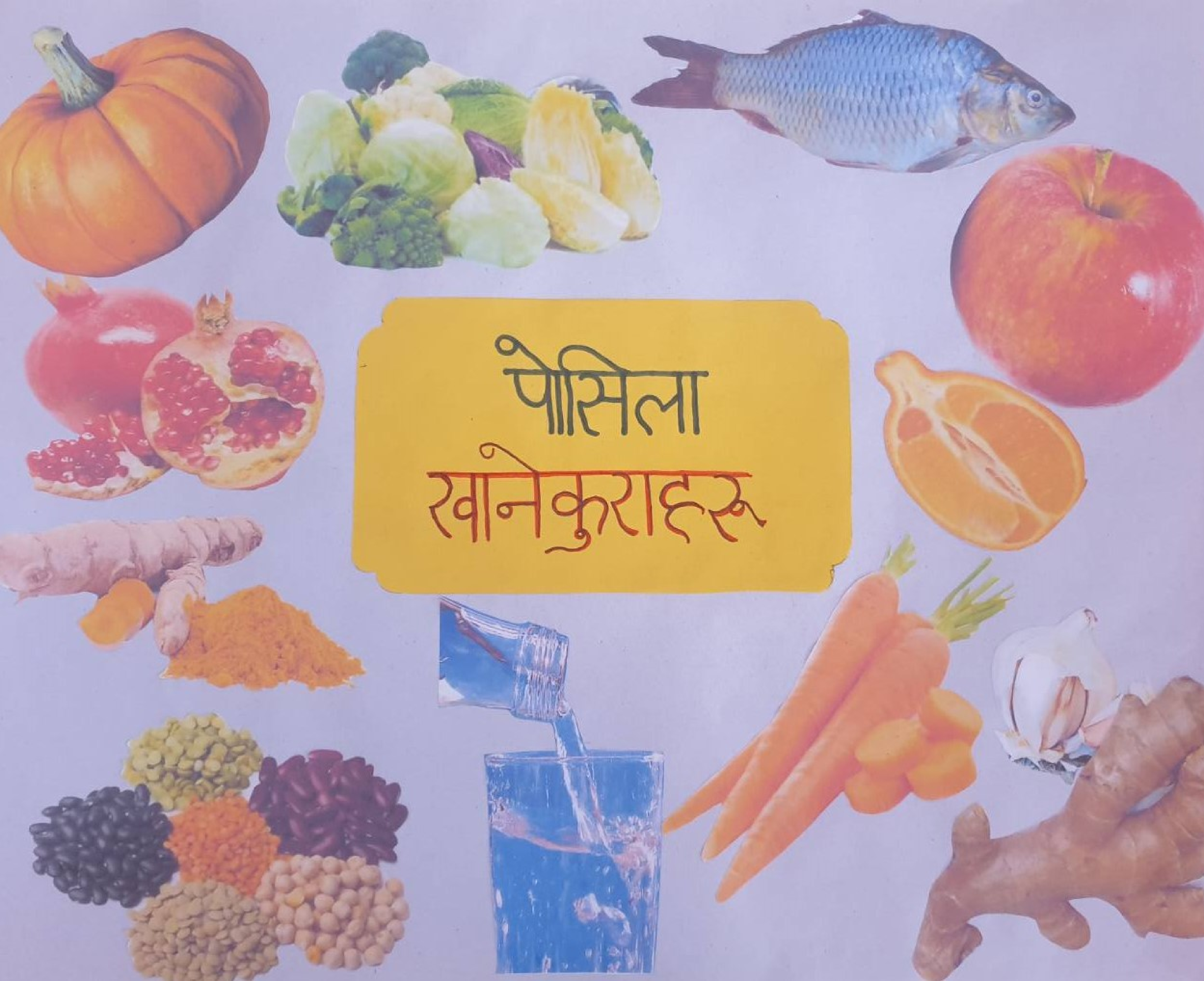
पोसिला
खानेकुराहरू

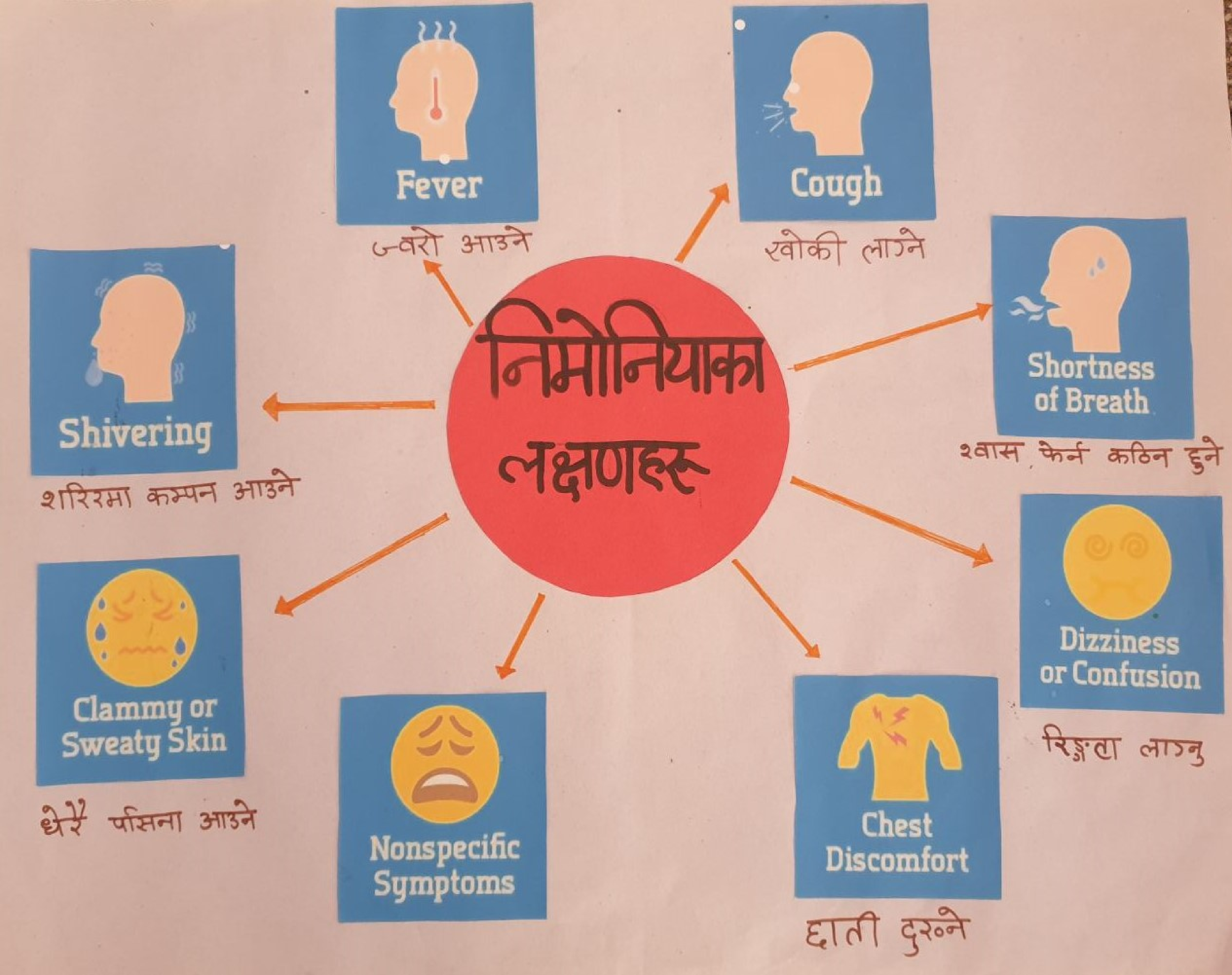
निमोनियाका लक्षणहरू
Fever
ज्वरो आउने
Cough
खोकी लाग्ने
Shivering
शरिरमा कम्पन आउने
Shortness of Breath
श्वास फेर्न कठिन हुने
Clammy or Sweaty Skin
धेरै पसिना आउने
Nonspecific Symptoms
Chest Discomfort
छाती दुख्ने
Dizziness or Confusion
रिङ्गटा लाग्नु
