# Supplementary material for: Effectiveness of childhood pneumonia awareness program among pregnant women: A hospital based quasi experimental study
Source: PLoS One. 2026 Nov 6;21(11):e0342216. doi: 10.1371/journal.pone.0342216 (PMC13742872; doi:10.1371/journal.pone.0342216)
Supplement: Supplementary file 2 [file pone.0342216.s002.pdf]

## S2 Appendix. Data Collection tools

**Socio Demographic**

| **Q.N** | **Questions** | **Response** |
|---|---|---|
| 1 | Age of respondent | |
| 2 | Ethnicity | 1. Brahmin /Chhetri<br>2. Madhesi<br>3. Dalits<br>4. Janajati<br>5. Muslim<br>6. Others |
| 3 | Religion | 1. Hindu<br>2. Buddhist<br>3. Islam<br>4. Christian<br>5. Kirat<br>6. Others |
| 4 | Family size | |
| 5 | Family type | 1. Nuclear<br>2. Joint/ Extended |
| 6 | Is this your first pregnancy? | 1. Yes<br>2. No |

| | | |
|---|---|---|
| 7 | If No, how many children did you give birth to ? | |
| 8 | Current Weeks of Pregnancy | |
| 9 | Education Status | 1. Illiterate<br>2. Literate<br>3. Primary School<br>4. Secondary School<br>5. Higher Secondary<br>6. Above higher secondary |
| 10 | Employment Status | 1. Self -employed / labour<br>2. Business<br>3. Service<br>4. Student<br>5. Home maker<br>6. Unemployed<br>7. Other |
| 11 | Place of residence | 1. Urban<br>2. Rural |
| 12 | Do you have experience of caring a child | 1. Yes<br>2. No |

**Pre - post test Questions**

1. What is Pneumonia? (Single response)
    A. Infection and inflammation of the lungs
    B. Infection and inflammation of heart
    C. Infection and inflammation of the pancreas
    D. Infection and inflammation of the kidney
    E. Don't Know

2. What age group are mostly affected by pneumonia? (Single response)
    A. Adults ages 65 and older
    B. Children younger than age 2
    C. People with certain medical conditions
    D. People that smoke
    E. Don't Know

3. What are the most common features of Pneumonia? (multi response questions)
    A. Fever
    B. cough
    C. chills
    D. fast breathing
    E. breathing with grunting or wheezing sounds
    F. working hard to breathe
    G. vomiting
    H. chest pain, chest indrawing
    I. belly pain
    J. being less active
    K. loss of appetite (in older kids) or poor feeding (in babies)
    L. Don't Know
4. How can Pneumonia be transmitted?
    A. With contaminated food and water
    B. With droplets from mouth and nose (sneezing and coughing)
    C. Transmission through faeces
    D. Transmission through fomites
    E. Don't know

5. Does Pneumonia transmitted from an infected pregnant woman to a foetus? (Single response)
    A. Yes
    B. No
    C. Don't Know

6. Is a vaccine available for Pneumonia? (Single response)
    A. Yes
    B. No
    C. Don't Know

7. Pneumonia can be prevented by Pneumonia vaccination? (Single response)

    A. Yes
    B. No
    C. Don’t know
8. If yes, how many times can it (vaccine) be taken? (Single response)
    A. 2 times

B. 3 times
C. 1 time
D. Don’t know

9. At what age does a baby can be vaccinated with Pneumonia vaccine in Nepal ? (Single response)
   A. Soon after birth
   B. 6, 10 and 14 weeks
   C. 6th, 10 weeks and 9 months
   D. Don’t Know

10. Do we need to pay for Pneumonia Vaccine for children in Nepal? (Single response)
    A. Yes
    B. No
    C. Don't Know

11. Is Pneumonia a curable disease? (Single response)
    A. Yes
    B. No
    C. Don't Know

12. Risk factors of Pneumonia in children? (Single response)
    A. Malnutrition
    B. Weakened immunity
    C. Pre-existing health illness(eg. Measles, HIV, Heart Diseases)
    D. All of the above
    E. Don't Know
13. Are the children exposed to second hand smoking at greater risk of pneumonia? (Single response)
    A. Yes
    B. No
    C. Don't Know
14. What are the environmental factors responsible for increasing the risk of pneumonia for children? (Single response)
    A. Air Pollution
    B. Secondhand smoking
    C. Poor environmental and physical hygiene
    D. All of the above
    E. Don't Know

15. What should we do, after recognizing the danger sign of Pneumonia in babies? (Single response)

A. Feeding home remedy as Tulsi, honey and ginger
B. Wait and see to recover baby ownself
C. Brought medicine from Medical store (pharmacy) and feed baby
D. Seek medical care as soon as possible
E. Don't Know

16. How satisfied are you with your current awareness level? (Single response)
    A. Highly unsatisfied
    B. Unsatisfied
    C. Satisfied
    D. Highly satisfied

**Qualitative Guide**

| **Main Questions** | **Sub questions** | **Probing** |
|---|---|---|
| Introduction | Briefly introduce about yourself | Age, pregnancy status, residency, education , employment, etc (Socio-demographic) |
| Knowledge related Question | What do you know about Pneumonia before taking an awareness session ?<br>What was the source of information | Sign and symptoms, mode of transmission preventive methods (vaccines, nutrition, etc) |
| | What do you know about Pneumonia after taking an awareness session ? | Sign and symptoms, mode of transmission preventive methods (vaccines, nutrition, etc) |
| | Does this awareness campaign increase your knowledge level?? | |
| Attitude related Questions | What was your attitude towards pneumonia disease before taking an awareness session?<br>How did you perceive the disease pneumonia?? | It's severity, treatable or not?? Vaccine availability |
| | What was your attitude towards pneumonia disease after taking an awareness session? | It's severity, treatable or not?? Vaccine availability |

| | | |
|---|---|---|
| | Does this program change your attitude towards Pneumonia ?? | |
| Effectiveness of Program | Is this awareness program effective for you ?? Why and How??? | |
| | What are your recommendation regarding awareness program | |
| | What are your recommendations regarding Pneumonia prevention and control programs? | |
| Ending Questions | If you want to add something in this interview you can add??? | |
